# First identification of a Neanderthal bone spear point through an interdisciplinary analysis at Abric Romaní (NE Iberian Peninsula)

**DOI:** 10.1038/s41598-024-67817-w

**Published:** 2024-08-19

**Authors:** Paula Mateo-Lomba, Andreu Ollé, Juan Luis Fernández-Marchena, Palmira Saladié, Juan Marín, M. Gema Chacón, Josep Vallverdú, Isabel Cáceres

**Affiliations:** 1 Institut Català de Paleoecologia Humana i Evolució Social (IPHES-CERCA), Zona Educacional 4, Campus Sescelades URV (Edifici W3), 43007 Tarragona, Spain; 2 Departament d’Història i Història de l’Art, Universitat Rovira i Virgili, Avinguda de Catalunya 35, 43002 Tarragona, Spain; 3 Departament de Prehistòria, Arqueologia i Història Antiga, Universitat de València, Avinguda Blasco Ibáñez 28, 46010 València, Spain; 4 Unidad Asociada al CSIC, Departamento de Paleobiología, Museo Nacional de Ciencias Naturales, C/José Gutiérrez Abascal, 2, 28006 Madrid, Spain; 5 Departamento de Prehistoria y Arqueología, Universidad Nacional de Educación a Distancia (UNED), Paseo Senda del Rey, 7, 28040 Madrid, Spain; 6 UMR-7194 – HNHP (MNHN – CNRS – UPVD – Sorbonne Universités), Muséum National d’Historie Naturelle, Paris, France

**Keywords:** Archaeology, Cultural evolution

## Abstract

Osseous industry has been observed at an increasing number of Neanderthal sites. Bone fragments were used for practical purposes, and a range of bone shaping techniques were employed. The variability of bone tools observed in different assemblages reflects considerable functional diversity. However, no bone spear points have been reported from these contexts. A comprehensive analysis of a bone spear point from the Middle Palaeolithic site of Abric Romaní (Barcelona, Spain) is presented. Through an interdisciplinary, multi-technique, and multi-scale approach combining technology, taphonomy, and functional analysis, compelling evidence for manufacture, use, and hafting was uncovered. The specimen exhibits clear signs of intentional knapping. The presence of microscopic linear impact marks, an impact fracture at the tip and potential internal stress fractures indicate its use as a spear. Furthermore, the observed wear pattern and a morphological adjustment of the trabecular tissue support the hafting hypothesis. Abric Romaní contributes to our understanding of Neanderthal hunting behaviour and the significance of composite bone tools in their technological repertoire 50,000 years ago. This discovery highlights the flexibility and adaptability of Neanderthal technology, providing evidence of bone technology that is sometimes obscured in the archaeological record and offering valuable insights into their hunting strategies during the Middle Palaeolithic.

## Introduction

Bone technology has been utilised by various hominin species since the Lower Palaeolithic. The earliest pieces of evidence are found in South Africa in Drimolen (ca. 2–1.5 Ma), Sterkfontein (Member 5, ca. 1.7–1.4 Ma) and Swartkrans (Members 1–3; ca. 1.8–1 Ma)^1,2^, as well as in East Africa, from Bed I to IV (1.8–0.8 Ma) in Olduvai Gorge^3–5^. In Europe, the presence of bone tools is also attested in Pre-Neanderthals sites such as Boxgrove (MIS 13)^6^, Gran Dolina (MIS 9)^7^, Castel di Guido (~ 400 ka)^8,9^, Isoletta, Colle Avarone, Selvotta (MIS 11–10)^10^, Bilzingsleben (412–320 ka)^11^, Vértesszölös (MIS 13–9)^12^, and Schöningen (478–424 ka)^13^, among others. There is also evidence for Neanderthals using bone tools as hammers or for retouching^14,15^ or as retouched or specialist tools^16–19^.

The manufacture of standardised forms shaped using more complex techniques like grooving, scraping or grinding is characteristic of the so-called formal bone tools in the Upper Palaeolithic. The bone industry of these earliest periods consisted mainly of unshaped bone fragments, considered to be the result of an opportunistic use of bone fragments^20–22^, minimally modified tools or artefacts shaped by percussion^9,23^ or grinding^1,24^. However, the functionality of these tools is not always clear. They are often attributed the same uses as the lithic artefacts they resemble^8^ and, with the exception of bone hammers and retouchers^13,25–27^, few studies integrate bone tool functionality into the Lower Palaeolithic^13,28–30^, Middle Palaeolithic^16,17^ or Middle Stone Age (MSA)^31–33^.

The investigation of use-wear traces on bone tools has been enriched by increased experimental studies^34–39^ and comparisons with ethnographic elements^40^. The aim of that work was to solve specific issues in each context studied, such as recognising expedient bone tools in the Lower Pleistocene in Africa^41,42^, assessing different past activities in the Middle Palaeolithic of East Asia^43^, in early sites in North America^44^, or formal bone tools in the Upper Palaeolithic-Epipalaeolithic in Levant^45,46^ or Holocene sites in South America^47,48^.

Specifically, the bone tools found in the Middle Palaeolithic archaeological record would have been used for a variety of activities that can be grouped mainly under the headings of knapping and maintenance of the stone industry, digging, woodworking and hide-processing. These would be actions involving percussion, drilling, scraping and so on, performed using retouchers/hammers and wedges, awls and smoothers, respectively^16,21,49–52^.

In ancient chronologies, pointed shapes are frequently linked to activities like perforations with awls or impacts with projectiles. To avoid any confusion, we prefer the broad definition of projectile and armature as synonyms from Rots and Plisson^53^, which includes thrown and thrust points. The presence of pointed bone elements in the Pleistocene archaeological record has led to debate about their origin, manufacture, and potential functionality^54^. In general, these are fully shaped objects manufactured through scraping or grinding, with a pointed distal end and recognisable by their morphometric characteristics, which are reminiscent of armatures used in later chronologies.

The increase in the production of formal bone tools, particularly bone weapons, took place during the Upper Palaeolithic^55–57^ and in the MSA, associated with anatomically modern humans. However, the earliest evidence is found in Africa, like the purported barbed point preform from WK East A in Olduvai Gorge (0.93–0.8 Ma)^5^, or the more likely tips of spears, specifically found in El Mnasra (Morocco) (ca. 107–106 ka BP)^58^ and Katanda (ca. 90 ka BP)^59^, but it also appears later in South Africa in Blombos Cave^31^, as well as the pre-Still Bay layers at Sibudu Cave dated to ca. 72 ka BP^1,60^. In Europe, although taphonomic studies have ruled out a large number of pseudo-tools^54^, isolated examples are known in Mousterian contexts from Salzgitter-Lebenstedt, Vogelhed, Große Grotte (Germany)^61–63^ and Divje babe I (Slovenia)^64^.

Moreover, the identification of prehistoric bone tools that have undergone impacts, likely due to hunting-related actions, has partly been addressed through macrofracture analysis^65,66^. Studies of distal fractures in the bone industry adapted the approaches used in lithic projectile analysis^67,68^. Nevertheless, the impact fracture terminations produced in hunting can be similar to other tip damage produced by other factors like trampling or accidental breakage^69^, as some experimental works have acknowledged^70,71^.

Another feature for identifying projectile components is the presence of hafting wear. Being hafted to a shaft is a necessary condition for projectiles. This element adds weight to the projectile and serves to save the (typically) wooden shaft from breakage during use. Moreover, it enables the target to be hit with greater force, increasing its penetration and cutting potential, and enabling it to be thrown from a safer distance^72,73^.

A number of lines of evidence suggest that Neanderthals may have hunted at close range, using stabbing spears—that could be thrown over short distances—and/or thrusting spears^74–76^. It is assumed that Neanderthals used lithic points as hafted spear tips^73,76–78^. Their projectile technology must have been attached to hand-held spears due to the characteristics of the lithic projectiles, but we cannot rule out the use of wooden weapons, which may not have always been preserved^79^. Recent ethnographic reviews have challenged the notion of Neanderthal hunting exclusively at close range. They suggest that hunting weapons might have been used in multiple ways, both as thrusting and throwing weapons, at different ranges, as evidence supporting one function does not preclude the use of another^80,81^.

**Figure 1:**
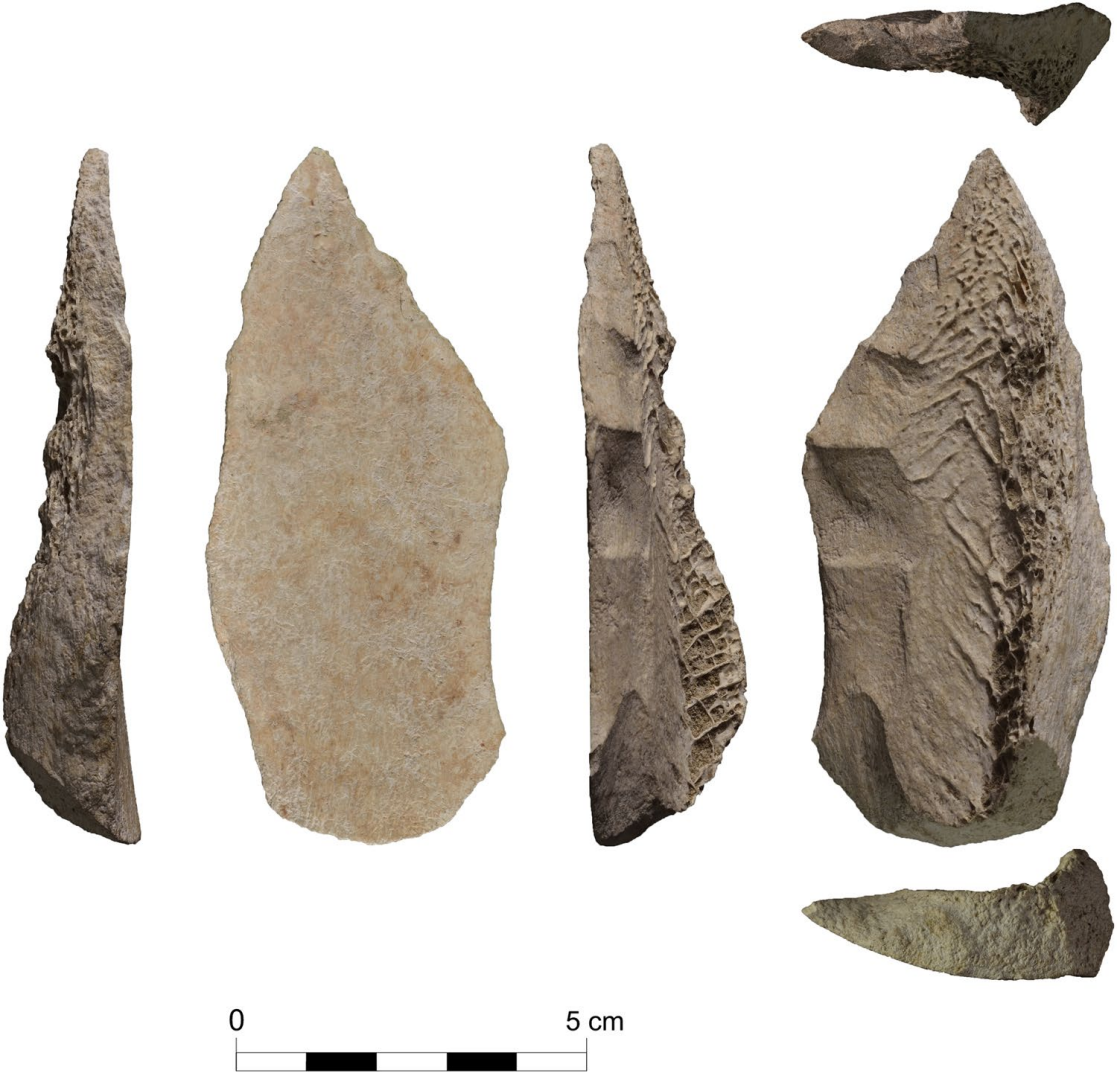
Different views of the described pointed bone.

The Abric Romaní site is a rockshelter located in the Cinglera del Capelló Gorge (Capellades, NE Iberian Peninsula), encompassing a predominantly Middle Palaeolithic stratigraphic sequence. More than 40 years of modern excavations have documented different types of occupation and subsistence strategies (see SI 1). Level Ja, interpreted as a residential place for human groups during long seasonal occupations^82^, yielded the specimen studied here (Fig. 1) with an interdisciplinary method that combined taphonomy, technology and traceology. Previous studies had already reported its existence^82^ but no hypotheses about its functionality had been proposed. Thus, this article aims to present evidence of the use of a bone spear point approximately 50 ka ago by the Abric Romaní Neanderthal groups and assess its significance in their hunting technology.

## Results

### Technological and taphonomic analysis

The long-bone shaft fragment was obtained from a large-sized taxa, probably an equid femur. The acquisition of the blank started with the butchery of the animal. Its cortical surface presents two groups of cutmarks—straight incisions arranged at an oblique angle—on the cortical surface, suggesting defleshing of the hindlimb (see SI 2, Fig. S11). According to the fragmentation analysis, the fracture planes are curved, smooth and oblique, indicating that bone breakage occurred when the bone was in a fresh state, to access the marrow and probably to obtain the blank.

From a technological point of view, the bone blank was unifacially shaped into a pointed form through at least three continuous removals on the right edge of the medullary surface, with a possible marginal removal towards the tip. The main removals are extensive, with a plane angle of retouching and a continuous disposition. The shaped edge is convex in frontal view and slightly sinuous in profile. The dimensions of the knapped bone are 93.2 × 41.8 × 14.1 mm and it has a cortical thickness of 9 mm.

**Figure 2:**
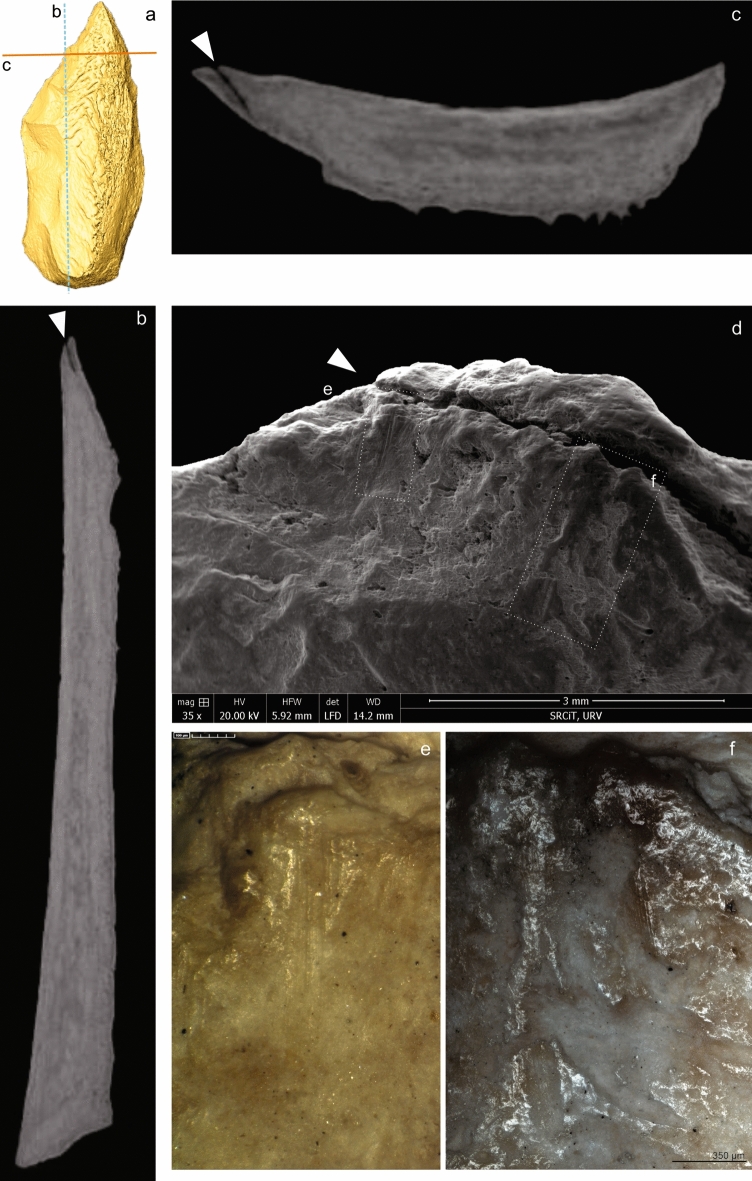
(**A**) 3D reconstruction of the described bone point. (**B,C**) Same crack from intentional shaping. (**D**) Fissure visible from the cortical surface, located on the opposite side to the medullary removal. Image obtained with low vac. SEM, secondary electron detector. (**E,F**) Sets of linear marks associated to the small adhering flake interpreted as technical traces. Images obtained with 3D digital microscope (**E**), and OM (**F**). Original magnification: 35 × (**D**), 600 × (**E**), 100 × (**F**). Scale bars: 3mm (**D**), 100 µm (**E**), 350 µm (**F**).

**Figure 3:**
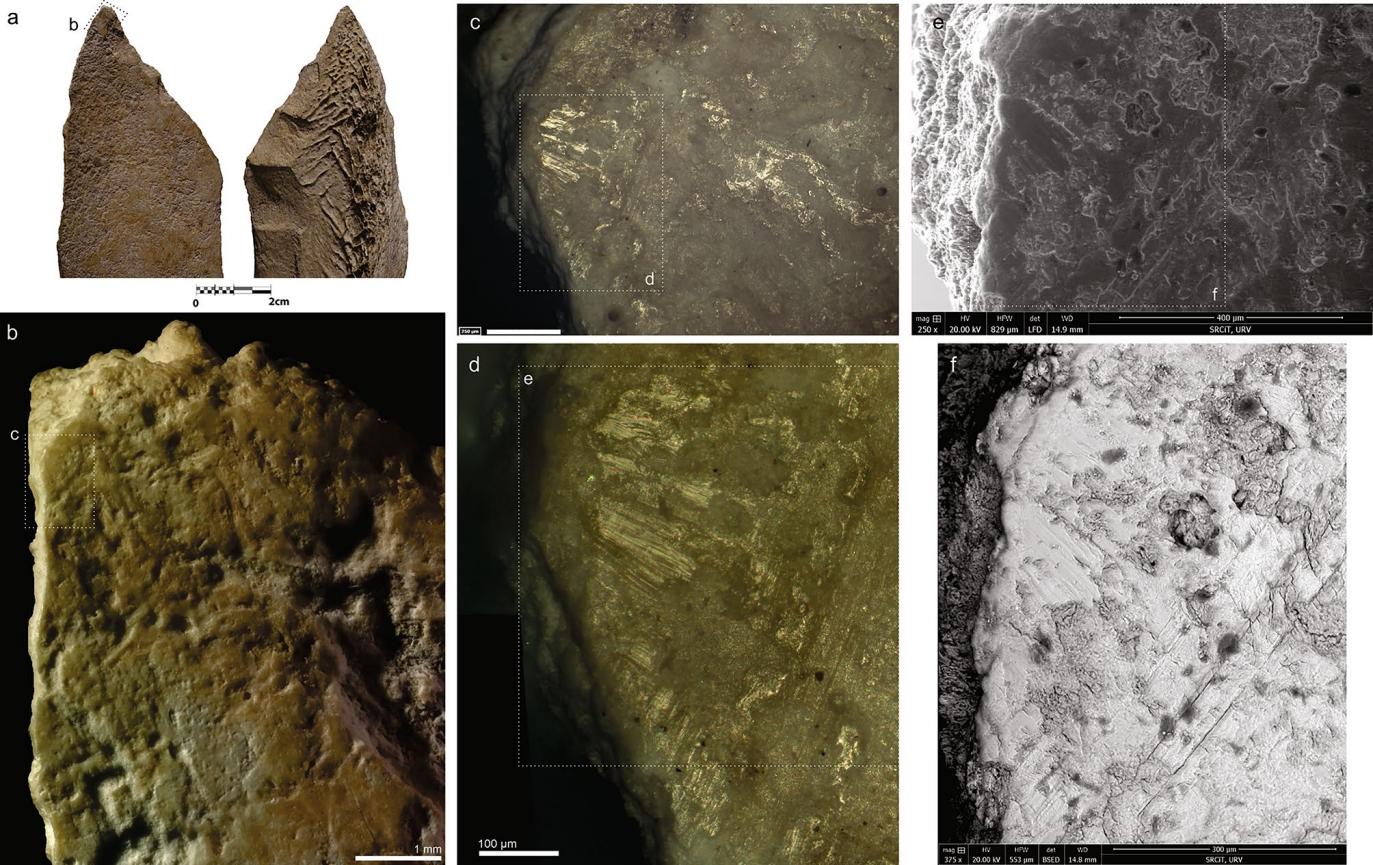
(**A**) Detail of distal part at cortical and medullary surface. (**B**) Localization of the use-wear associated with the tip fracture. Images obtained with 3D digital microscope (**B–D**), and SEM (**E,F**). Original magnification: 35 × (**A**), 140 × (**C**), 600 × (**D**), 250 × (**E**), 375 ×(**F**). Scale bars: 2 cm (**A**), 1 mm (**B**), 250 µm (**C**), 100 µm (**D**), 400 µm (**E**), 300 µm (**F**).

In addition, there is an incipient removal towards the medullary face that is visible from a crack on the cortical one, which can be followed internally (Fig. 2; the locations of the microphotographs, Figs. 3, 5, 6, S13, S14, S15, S16, can be found in Fig. S12). Some groups of parallel striations appear perpendicular to the edge and in the opposite position to one of the removals; these are interpreted as retouch traces. This interpretation is supported by analogues in experimental knapped bone tools^39^.

There are some peeled areas on the cortical surface, probably not related to the shaping phase, but due rather to post-depositional events. Moreover, root-etching is present on both cortical and medullary surfaces, but this is more generalised and invasive on the cortical surface. Trabecular structure is also present on the right part of the medullary surface running longitudinally but this is interrupted by a transverse channel near the tip (Fig. S12).

### Functional analysis

**Figure 4:**
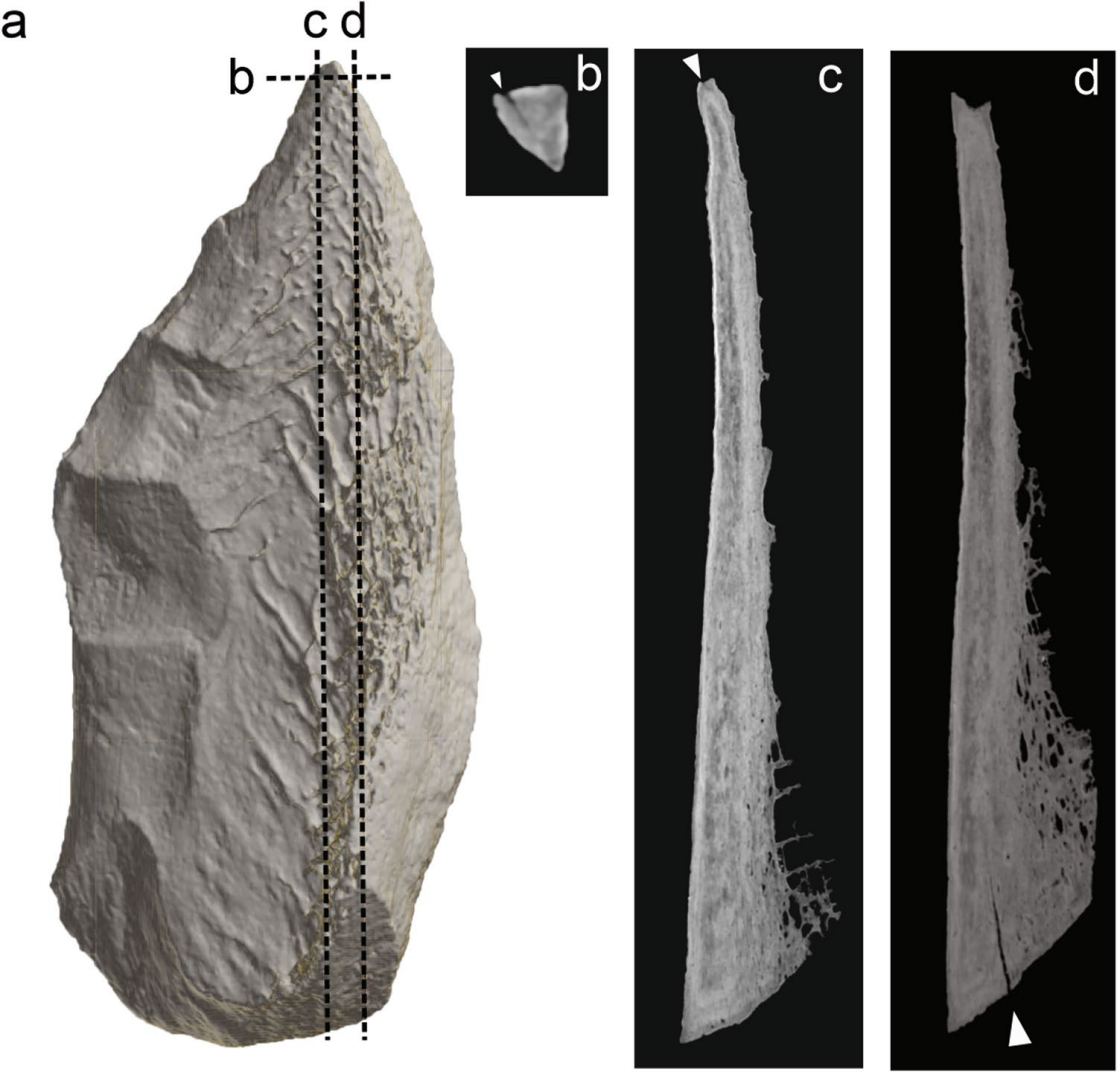
(**A**) Medullary surface from the 3D reconstruction. (**A,B**) Crack associated with the fractured tip. (**B**) Internal crack in the proximal area. (**A**) µCT horizontal slice, (**B,C**) µCT sagittal slice.

Traceological analysis provided use-wear and hafting evidence for the Abric Romaní knapped bone tool. The clearest sign of use is a distal fracture. The tip damage can be described as a step-terminating bending fracture (Figs. 3A, B, and Fig. S13A). On the tip there is a macrofracture related to a possible stress crack in the same area. The micro-CT slices also show that there is a single internal fissure starting at the tip, oriented obliquely with respect to the axis of the piece. This can be seen in the longitudinal and transverse sections (Fig. 4B–D) and is similar to those present in pointed bone projectiles^83–85^.

In addition, on the cortical surface of the tip we found a linear band of polish^86^ oriented obliquely to both the longitudinal axis of the specimen and the edge (Fig. 3 and compare with SI 3 and Figs. S22, S24, S27, S29, S32). It is very similar to the microscopic linear impact traces (MLIT) described in lithic projectiles^67,87^ associated with tip damage. This type of trace was found only in this area of the tool. It does not resemble other use-wear marks produced during cutting, scraping, or drilling activities^39,47,88^. This mark is interrupted by a linear mark of possible post-depositional origin.

On the medullary face, as well as on the tip, there is some polish in the upper areas where several groups of parallel linear marks can be observed (Fig. S13D–K and compare with Figs. S24, S25 and S32). According to Rots^87^, and making a parallelism with stone tools, these could have been produced by the scar flake that detached upon impact and briefly scratched the surface. As a result, these marks start at the termination of the impact scar or fracture, and are always oriented (roughly) parallel to the axis of use. There are no other signs of use-wear in this distal part (Fig. S16).

**Figure 5:**
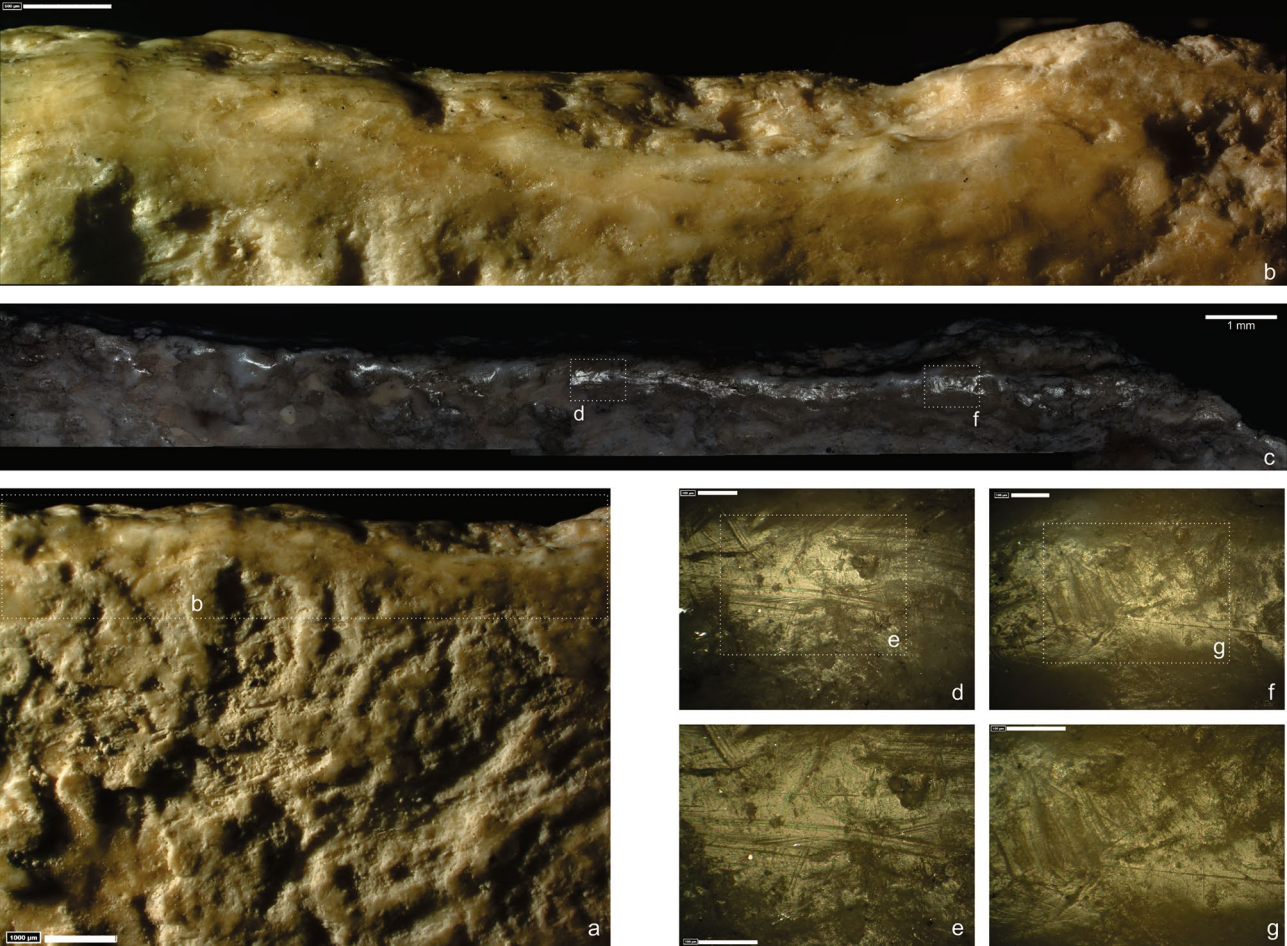
(**A**) Localization of the flattened band on the right side of the tool from the medullary surface. (**B,C**) Flattened area. (**D–G**) Details of the polished areas and randomly oriented linear marks. Images obtained with 3D digital microscope (**A–B,D–G**), and OM (**C**). Original magnification 35 × (**A**), 140 × (**B,C**), 400 × (**D,F**), 600 × (**E,G**). Scale bars: 1000 µm (**A,C**), 500 µm (**B**), 100 µm (**D–G**).

On the left edge, on the medullary face of the tool, a longitudinal flattened area parallel to the edge can be seen at low magnifications. When zoomed in, this area shows groups of randomly oriented linear marks (Fig. 5 and compare with Fig. S25).

In terms of evidence correlating with hafting, the retouched edge displays slight rounding, both on the cortical and medullary sides, and lateral scarring only on this edge of the distal part of the tool.

On the medullary surface of the right edge there is a similar pattern, with parallel linear marks oriented perpendicular to the edge. There are also some small-scale scars close to the edge (Fig. S14 and compare with Figs. S23 and S28).

**Figure 6:**
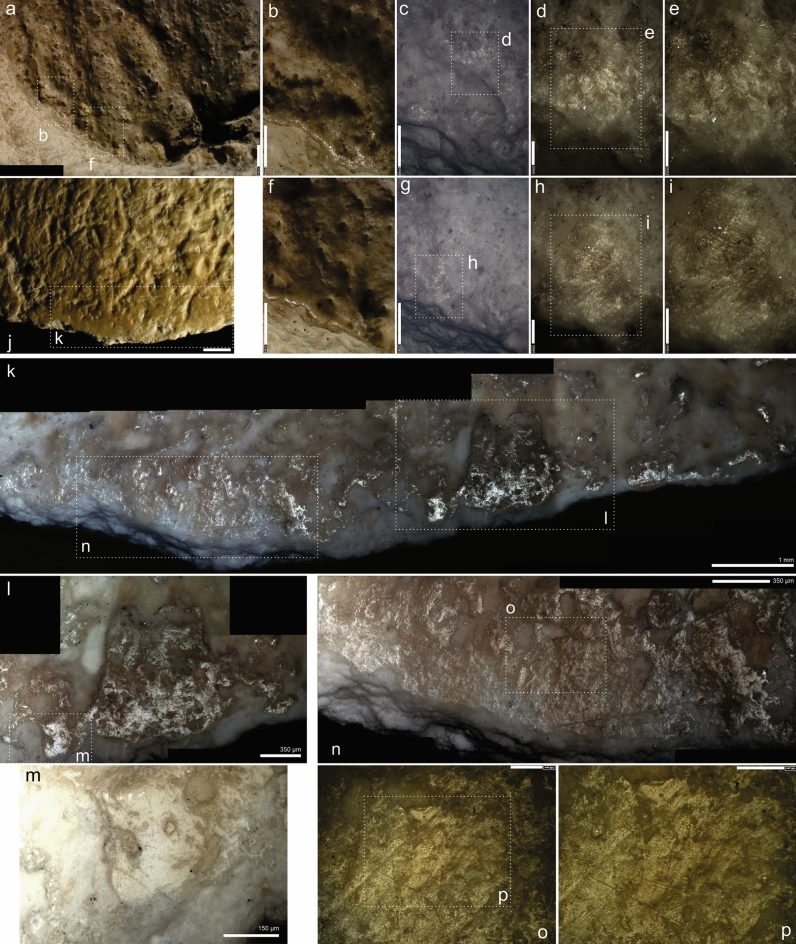
(**A**) Proximal end of the tool at the medullary surface. (**B–E**) Rounding of the edge and polished observed at medullary surface. (**F–I**) Rounding of the edge, polishing associated with linear marks, randomly oriented. (**J**) Proximal part of the tool viewed from the cortical surface. (**K**) Localization of the use-wear close to the edge. Polished surfaces appear associated with crossed, randomly oriented linear marks. Images obtained with 3D digital microscope (**A–J,O–P**), and OM (**K–M**). Original magnification: 35 × (**A**), 140 × (**B–C,F–G**), 400 × (**D,H**), 600 × (**E,I**). Scale bars: 1000 µm (**A,J,K**), 500 µm (**B,C,F,G**), 350 µm (**L,N**), 150 µm (**M**), 100 µm (**D,E,H,I,O,P**).

Other marks related to the hafting were identified on the proximal part of the tool. On its cortical surface, on the right side, there is a group of parallel linear marks perpendicular to the edge (Fig. S15). Towards the bottom, there is a concentration of polish and striations in an area unaffected by the root-etching, which affects the preservation of the tool’s surface (Fig. 6J–P and compare with Fig. S33). The striations follow a cross-oriented and random distribution, and are heterogeneous in their morphology, although some of them are perpendicular to the edge, similar to the hafting traces described by Buc^47^. In the same area of the medullary surface, there are some areas with striations showing clear parallel patterns (Fig. 6A–I).

There are no visible cracks at the base, although micro-CT scanning revealed an internal crack in this area that could have been caused during the manufacture or use of the artefact (Fig. 4D). This is likely to be damage produced by the thrust of the handle at the moment of impact with the target^65^.

## Discussion

A tool can be considered a hunting weapon if its function can be convincingly argued through diagnostic impact fractures (DIFs) and MLITs on the tip, in association with other signs of use, such as hafting traces, and if it has the appropriate morphology to function as a component of an armature^53,54,77^. The interdisciplinary method and multi-technique analysis undertaken here revealed a range of evidence consistent with the production and use of a hafted bone spear point.

To make this spearhead, the toolmakers used a common element among the taxa nutritionally exploited by the Neanderthals who occupied the Ja sublevel at Abric Romaní^82^. It is therefore not a specific selection. The intensive exploitation of marrow at various levels, and specifically in Level Ja^89^, suggests its prior use for nutrients, especially bone marrow. However, we cannot discern whether the bone blank was obtained from bone fracturing for nutritional purposes and its shaping was part of an opportunistic exploitation or whether it was intentionally obtained as a blank for technological purposes.

At Abric Romaní, specifically in sublevel Ja, the variability of lithic raw materials and the abundance of artefacts of all sizes is remarkable^90^. In this sense, we can rule out the hypothesis that the scarcity of suitable lithic raw material supports would have meant these human groups resorted to other raw materials, such as bone, for technological exploitation. This explanation has been suggested for other contexts where knapped bone industry has been found^8,11^. Thus, the use of a fragment of diaphysis as a raw material is in line with the flexibility observed in the technological behaviour of the lithic assemblage.

There is intentionality in the manufacture and shaping of the tool. The continuous removals configured a pointed shape in the distal part of the bone tip. Other studies reported limited shaping through direct percussion in sites including Chez-Pinaud^19^, Chagyrskya^51^, La Quina, Bois-Roche^49^ and Fumane Cave^17^. Therefore, the general morphology of the specimen, the presence of the removals, their disposition and the technical marks identified on the surface allows us to define the specimen as a knapped bone tool.

Furthermore, based on the morphometric characteristics and the nature and distribution of the use traces, we can propose that this bone tool was used as a hafted spear point. The functional specificity and diversity observed in other Neanderthal bone tool assemblages, i.e., lithic tool making, woodworking and hide-processing^14,16,17,19,20,51,52,91^ is, in some cases, extended with the results obtained from the analysis of this specimen.

The MLITs identified on the cortical surface (Fig. 3), together with the tip fractures, are considered diagnostic of projectile use in lithic studies^92,93^, but to our knowledge have not been described in osseous projectiles. Furthermore, there appears to be a macrofracture at the tip (Fig. 3 and Fig. S13) which is consistent with the tool being used as a projectile point^65^. In the case of level Ja, no projectiles were identified among the lithic assemblage^90^.

Hafting is essential in the case of projectiles or spearheads, unlike tools for butchery or woodworking, which can be handheld. Microscopic analysis has not revealed any embedded shaft remnants, adhesives or ligature systems in the form of residue. However, other evidence has been found^87^ that this bone tool would have been hafted. Based on the wear patterns described above on the hafted part, we could propose a hafting arrangement. The shaft would have rested in the medullary canal and also on the cortical surface. This suggests that the distal end of the spear would have been U-shaped to accommodate the base of the tip. The shaft would have been supported on both the cortical and medullary surfaces, as indicated by the marks preserved at the proximal end of these areas (Fig. 6).

A ligature system would connect this shaft to the bone point at the distal end of the tool, close to the tip where the lateral scar, the rounding (Fig. S14), and the distribution of flattened areas, with linear marks presenting preferred orientations on both sides (Fig. 5) are documented. The resulting cordage wear is consistent with that described in other works^47,94^. In addition, the morphological adjustment in the form of a groove in the trabecular tissue would have facilitated the attachment of this ligation system (Fig. S12). At the proximal end of the tool on the right-hand side of the cortical face, there are further striations that could be related to the ligation system in this area of the tool, but the presence of surface post-depositional modifications may have obliterated some of the binding marks (Fig. S15).

Pointed artefacts could fracture as a result of different sources of damage, including trampling and accidental falls^70,71^. The examination of the internal structure of the tool indicates possible stress fractures in the tip and base (Fig. 4). Previous studies based on experimental and archaeological evidence^65,83,85,95^, have shown that bone projectiles can develop microfractures due to impact loading that are not externally visible. In hafted tools, micro-cracks develop in areas of stress concentration, such as the tip and the proximal part of the tool^83,95^. However, such damage appears to be different from the aforementioned micro-cracks^83^.

Regarding the origin of the internal cracks mentioned above, it cannot be completely ruled out that the crack at the base (Fig. 4D) may have originated during the manufacturing phase of the tool or during the percussion fracturing of the bone. Exposure to a heat source is another factor that can cause internal cracking. Backwell et al.^95^ compared internal cracks caused by projectile impact with those resulting from exposure to a heat source—such as the hearths at the Abric Romaní site. The former generally run parallel to the planes of the histological channels while the latter show radial, centripetal cracks in transverse section and a jagged profile of microcracks in longitudinal section. They are also randomly distributed throughout the artefact and are more abundant than the former. Based on their location, arrangement and profile, the internal crack found at the tip on the Abric Romaní point could be consistent with impact.

The Ja sublevel has been interpreted as a seasonal camp specialising in deer and horse hunting. The activities carried out by the Neanderthal groups would have been related to complete butchering processes, hide-processing, the knapping of stone tools and the use of wood for domestic purposes^82^. The data obtained from the study of the lithic assemblage suggests manual prehension in its use^90^. However, the bone point discussed here provides new evidence of hafted and composite tools. The identification of a hafted portion and a hafting arrangement indirectly implies that these Neanderthal groups probably used spears for hunting.

The mortality profile, with prime adults among the equids and equal proportions of red deer juveniles and prime adults, suggests selective and non-selective hunting tactics, respectively, related to ambushes, likely combined with stalking. In these cases, cooperative strategies are the most common, even if the hunt is undertaken by a single individual^96^. These hunting tactics can be favoured by the use of spears^72^. This aligns with other indicators related to wood technology within this and other levels at the site, thereby introducing new questions into this subject matter^97^. Lithic or bone points were typically attached to shafts (usually made of plant or animal material). This served to increase the weight of the projectile and to protect the wooden shaft from breaking during use. Additionally, this allowed for greater impact force and improved the penetration and cutting potential of the target, all from a safer distance^72,73^.

The presence of this hunting weapon opens new lines of analysis into the presence or absence of projectiles in the Middle Palaeolithic record. The uniqueness of this bone artefact, being manufactured in an environment with available lithic raw material and its use as an armature, is not related to the technological skills of the Neanderthals from the Ja level. The isolated presence of this object may reflect the fact that the hunting tools used did not necessarily reach the residential camps, as they may have remained at the kill site^98^. The appearance of this used point could be understood in two ways: either it arrived still attached to the spear and was discarded, or it arrived inside the body of a hunted animal^77^.

## Conclusion

We have applied an interdisciplinary and multi-technique method to the study of a Middle Palaeolithic bone weapon. By combining an analysis of bone surface modifications with the internal damage, the applied method sheds light on an activity that was archaeologically invisible in this context and offers a reliable identification of a knapped bone tool used, probably, as a thrusting and/or throwing bone spear point. The wear pattern as a whole supports the interpretation, as do the impact fracture, morphometric characteristics, internal fractures, and results obtained from experimental testing. However, the evidence presented in this paper is not sufficient to propose a hypothesis about the delivery mode of this composite weapon. We are aware that the study of projectile elements is still extremely complex and requires adequate control of a number of variables, and it would obvious benefit from larger archaeological parallels.

Bone industry was used by Neanderthal groups at Abric Romaní and extends our knowledge of both their hunting technology, through their technical skills for shaping and using bone as a raw material, and their hunting behaviour, through the early occurrence of organic spear armatures and bone projectiles. This research paves the way for future investigation in the growing field of early bone technology, offering the potential to uncover additional insights into resource use, technological behaviour, and the subsistence strategies of ancient human groups.

## Methods

### Fragmentation analysis

8149 remains were analysed in a search for bone tools. Among them, 1328 long bones were studied according to bone breakage analysis criteria. The length of the shaft (L1: < 1/4; L2: between 1/4 and 1/2; L3: between 1/2 and 3/4; L4: > 3/4 of the total length) and shaft circumference (C1: < 1/2; C2: > 1/2; C3: complete circumference) were noted. Moreover, the outline (longitudinal, transverse, curved), angle (right, oblique, mixed), and fracture edge (smooth, jagged) of each fracture plane were considered; along with any bone surface modifications in the assemblage caused by intentional breakage, including percussion marks, notches, adhering flakes, cortical and medullary scars, and the presence of cortical and medullary flakes^99–103^. Morphometric data (length, width, and thickness) were measured using a digital calliper.

### Technological, taphonomic, and functional analysis

The identification of this knapped tool was based on a comparison with other archaeological and experimental specimens^5,9,17,23,24,32,36,42,49,104^ and our own experimental sample of fractured bovine bones, simple and knapped bone tools^39,105^.

The technological criteria for describing these are given in Mateo-Lomba et al.^105^, and involve an adaptation of the Logic Analytic System^106^ to the technological studies of bone industry.

Both natural taphonomic modifications^107–114^, technological and other anthropogenic traces^39,115,116^, such as use traces, are described according to the terminology used in previous works^17,35,39,47,88,117,118^.

The macroscopic documentation was undertaken using a digital camera (Nikon D780 with 40 mm AF-S DX Micro NIKKOR 40 mm f/2.8G objective). In addition, to improve the documentation of this artefact we scanned it using a structured light scanner (Artec Space Spider) and Artec Studio Professional (Artec Inc., Luxembourg; version 15). The 3D model is available in the Supplementary material.

The traceological analysis was conducted using a multi-technique and multi-scalar approach^39,119–121^. The functional analysis was performed directly on the specimen, without the use of replicas. The equipment used was a 3D digital microscope (Hirox KH-8700, MXG- 5000REZ Triple Objective), an optical microscope (OM; Zeiss Axio Scope A.1) from IPHES (Tarragona, Spain) and a Scanning Electron Microscope (SEM, FEI-QUANTA 600), used at Low Vacuum mode, from the Scientific & Technical Resources Service at Universitat Rovira i Virgili (Tarragona, Spain). Wear distribution was documented using the free image stitching software JOIN^122^ and the 3D tiling feature of the 3D digital microscope.

Sample preparation for microscopic analyses consisted of basic cleaning to accurately observe the active edges and their microscopic characteristics. The cleaning protocol consisted of washing in an ultrasonic bath containing a solution of water and lab detergent for 1–2 min (Derquim® 2%).

Damage from projectile impact has been distinguished from other agents^69^. Different tip fractures have been observed in both archaeological and experimental materials. Nevertheless, projectile identification is partly based on ‘diagnostic impact fractures’ (DIFs). Impact fracture analysis and associated use traces have been classified into general types following the literature on lithic projectiles^67,69^ and work on projectile osseous industry^65,66,71,123^. Besides fractures, the main types of impact marks are microscopic linear traces (MLITs)^92^ and tip damage. MLITs are linear marks transverse to the impact zone (generally longitudinal to the long axis of the projectile) and are defined as a mixture between polishing and linear marks. They mark the direction in which the detached fragments were dragged on impact, and therefore the direction the piece entered the target. The tip damage was described according to the terminology used in studies of osseous projectiles^65,66^. We also considered the presence, type and distribution of these marks on the osseous armatures.

Based on the results of an initial multi-technique microscopic analysis of the bone tool, a dedicated experimental programme was set up to improve our knowledge of impact and hafting traces, including a set of unshaped pointed bones used as hafted spear points in thrusting actions (see Supplementary material 2).

In addition to microscopic examination of the surface of the archaeological bone tool, we also examined its structure using micro-CT scanning to gain insight into possible internal damage. The images were taken with a model V| Tome|X s 240 by GE Sensing and Inspections Technologies from CENIEH (Burgos, Spain) with a scanning energy of 160 kV and 250 µA. A total of 1800 slices were acquired. A 0.2 mm Cu filter was used together with a voxel size of 0.06 mm^3^ [this data is available at Zenodo [https://doi.org/10.5281/zenodo.10808292)]. By means of longitudinal, transverse, and sagittal sections, this technique allows the observation of internal cracks or fissures and the rendering of a 3D reconstruction of the scanned piece. The 3D image was obtained using Amira 5.2 software. The interpretation of the internal micro-cracks is based on previous publications^19,83,124,125^.

## Supplementary Information

Supplementary Information.

Supplementary Video 1.

Supplementary Video 2.

## Data Availability

All data are presented in the main text and the Supplementary Information. Raw data from micro-CT scanning is available in the Zenodo repository at https://doi.org/10.5281/zenodo.10808292.
