# Supplementary material for: First identification of a Neanderthal bone spear point through an interdisciplinary analysis at Abric Romaní (NE Iberian Peninsula)
Source: Sci Rep. 2024 Aug 19;14:19160. doi: 10.1038/s41598-024-67817-w (PMC11333748; doi:10.1038/s41598-024-67817-w)
Supplement: Supplementary file 1 — Supplementary Information. [file 41598_2024_67817_MOESM1_ESM.pdf]

Supplementary Information

First Identification of a Neanderthal Bone Spear Point Through an Interdisciplinary Analysis at Abric Romaní (NE Iberian Peninsula)

Paula Mateo-Lomba[a,b*], Andreu Ollé [a,b], Juan Luis Fernández-Marchena [c,a], Palmira Saladié [a,b,d], Juan Marín [e,a,f], M. Gema Chacón [a,b,f], Josep Vallverdú [a,b,d], Isabel Cáceres [b,a]

[a] Institut Català de Paleoecologia Humana i Evolució Social (IPHES-CERCA), Zona Educacional 4, Campus Sescelades URV (Edifici W3), 43007 Tarragona, Spain

[b] Universitat Rovira i Virgili, Departament d'Història i Història de l'Art, Avinguda de Catalunya 35, 43002 Tarragona, Spain.

[c] Departament de Prehistòria, Arqueologia i Història Antiga. Universitat de València. Avinguda Blasco Ibáñez 28, València, Spain.

[d] Unidad Asociada al CSIC, Departamento de Paleobiología. Museo Nacional de Ciencias Naturales, C/ José Gutiérrez Abascal, 2, 28006, Madrid, Spain.

[e] Departamento de Prehistoria y Arqueología, Universidad Nacional de Educación a Distancia (UNED), Paseo Senda del Rey, 7, 28040, Madrid, Spain.

[f] UMR-7194 – HNHP (MNHN – CNRS – UPVD – Sorbonne Universités), IPH, 1 Rue René Panhard, 75013 Paris & Musée de l'Homme, 17 Place du Trocadéro, 75016 Paris, France.

Corresponding author email: pmateolomba@gmail.com

Contents

1. Supplementary Information: The Abric Romaní 3
   Stratigraphy, sedimentology and paleoambient 7
   Archaeological record from level Ja 10
      Lithic assemblage 10
      Macrofaunal remains and taphonomy 14
      Hearths 16
      Archaeobotanical assemblage 19
2. Supplementary Information: Extended data about bone spear point found at Abric Romaní 20
3. Supplementary Information: Experimental test 26
   Bone points 26
   Spears and hafting technique 27
   Spear thrusting 27
   Functional analysis 30
   Description of the experiment and results 30
   Final remarks 43
   Ethics statement 44
SI References 44

1. Supplementary Information: The Abric Romaní

The Abric Romaní site is a wide rockshelter located in a travertine cliff called the Cinglera del Capelló in the town of Capellades (Barcelona, Spain) on the west bank of the Anoia River, 50 km west of Barcelona (Fig. S1) and 265 metres above sea level. The cliff escarpment is oriented NW–SE with the entrance on the NE side of the wall, facing the Capellades Gorge. Its coordinates are 18º41'30'' longitude E and 41º32' latitude N.

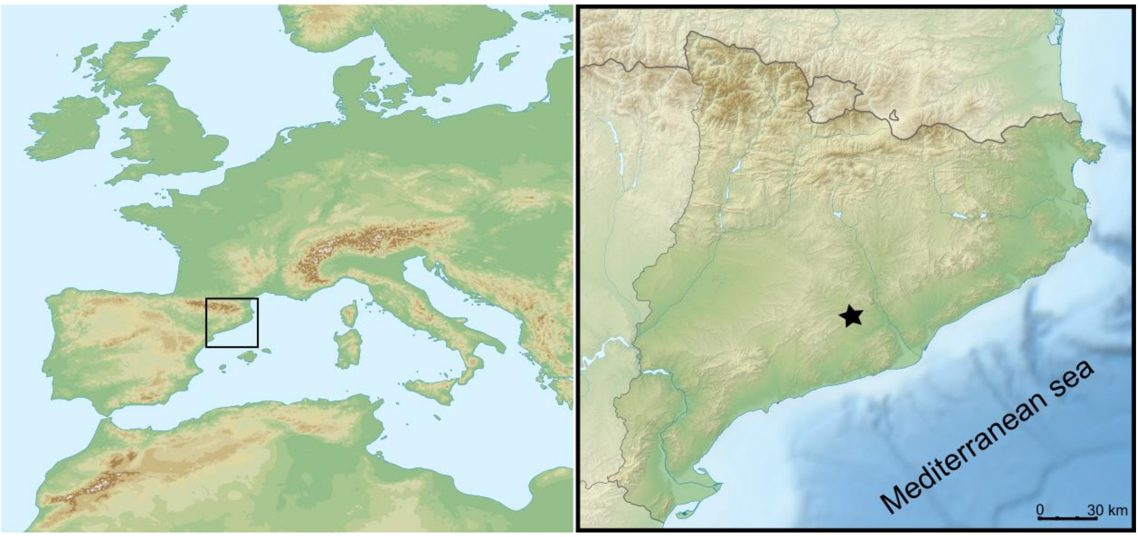

Figure S1. Location of Abric Romaní in northeast Iberia.

The Cinglera del Capelló has a long history of more than 100 years of research focused mainly on the Abric Romaní site. There is no doubt that the profound knowledge of the archaeological wealth of the Cinglera del Capelló is due to the figure of Amador Romaní i Guerra (1873-1930), dating back to the first third of the 20th century[1]. Romaní brought to light many shelters containing archaeological sites. Most of these contained materials belonging to the Neolithic and the Bronze Age. Others of these shelters provided materials from the Middle and Upper Paleolithic. The two most significant were the Agut Station and the one known at that time as Bauma del Fossar Vell, which was later renamed Abric Romaní. These shelters were extensively worked on by Amador Romaní. According to his descriptions on August 9, 1909, archaeological records were discovered at the Abric Romaní, which were reported to the authorities. From that moment on, the work was directed by Norbert Font i Sagué until he died the following year. Then, until 1911, the direction was carried out by Lluís Maria Vidal. Always under the supervision of Amador Romaní i Guerra. The work carried out during this period was sponsored by the Institut d'Estudis Catalans (ICE). After the sponsorship of the ICE ended, starting in 1912, it was Amador Romaní himself who financed and directed the excavations until 1930. His work affected much of the surface of the Abric, almost entirely carrying out the

excavation of layer 2 (now known as level A). Conversely, his interest in the stratigraphic sequence of the site led him to excavate a well (Pou 1) in order to reach the base of the deposit. This well was the deepest but not the only one he carried out[1].

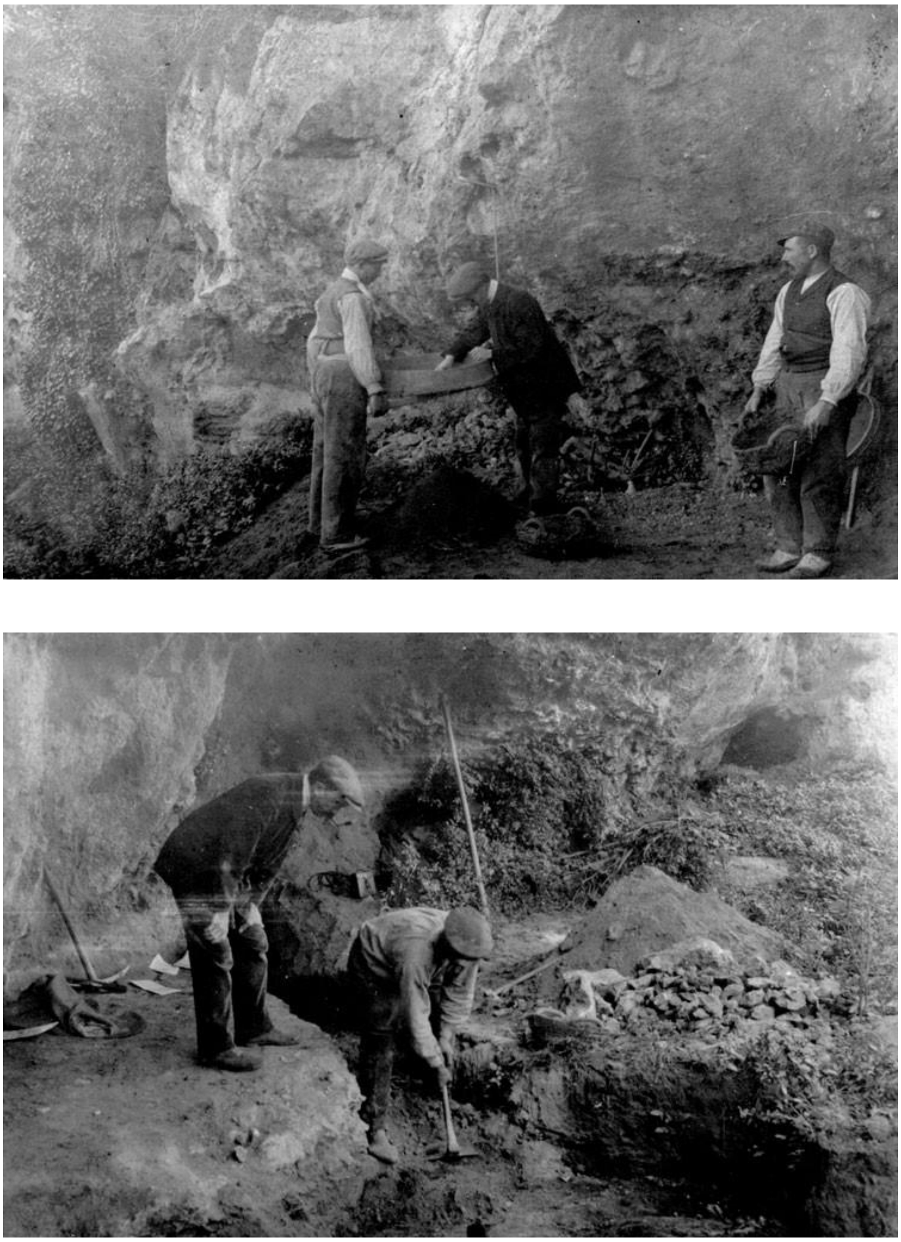

Figure S2. Amador Romaní (in the center of the image) during the excavations at the Abric Romaní in 1909. (Image: Arxiu Museu Molí Paperer de Capellades).

The Spanish Civil War (1936-1939) profoundly disrupted archaeological activities. This "dark period" persisted until the mid-1950s, when the end of the international isolation of the Franco regime favoured the reactivation of archaeological work, often associated with the collaboration of foreign scholars[2]. In this context, the second period of research at the Cinglera began in the mid–20th century. The V INQUA Congress held in Spain in 1955 included a visit to Abric Romaní led by the Archaeological Research Service of

Barcelona Provincial Council to promote these excavations, which finally began in 1956 under the direction of Dr Eduard Ripoll Perelló. The main objective of these interventions was to verify the stratigraphic sequence outlined by Amador Romaní [3]. The fieldwork was partly carried out by French researchers including Georges Laplace (who studied and published a description of the Upper Palaeolithic assemblage recovered by Romaní in layer 2[4] and Henry de Lumley.

The last historical period of interventions at the Cinglera dates back to the 1980s. In 1983, the project at the Cinglera del Capelló was resumed by a working group linked to the Autonomous University of Barcelona and the Palaeoecosocial Research Centre (CRPES) under the direction of Dr Eudald Carbonell, Artur Cebrià, and Dr Rafael Mora. The research programme in this latest phase follows an approach based on the extensive excavation of very thin and apparently well-preserved archaeological levels[5].

The excavation and research team from the Rovira i Virgili University (until 1992 the Tarragona delegation of the University of Barcelona) in Tarragona, began under the direction of Dr Eudald Carbonell and Artur Cebrià in 1989. Currently, this research team continues its work as part of the Catalan Institute of Human Palaeoecology and Social Evolution (IPHES-CERCA), currently directed by Dr Eudald Carbonell, Dr M. Gema Chacón, Dr Josep Vallverdú, and Dr Palmira Saladié. The research and development of these excavations have focused on the spatial documentation of structures and archaeological materials.

The extension intervention has covered 11 archaeological levels (from H to R), and 3 monographs dedicated to levels H, I, and J have been published [6–8]. Starting from archaeological level K, the extension intervention is complete and not affected by the probes from previous phases of archaeological work. The thickness of the sediments excavated is approximately 12 metres. A mechanical core has enabled another 30 metres of deposits from level P to be documented, which is about 9 metres below the last travertines of the shelter. The accumulated talus at the foot of the shelter's cornice is therefore at least 40 metres thick. This thickness means the base of the talus at the foot of the shelter's cornice is 260 metres above sea level. This elevation of 260 metres is very close to the elevation of the terrace of +20–25 metres embedded in the current bottom of the Anoia River valley[9].

Level J is one of the richest in the sequence, both in the quantity of archaeological remains uncovered there and in the number of occupation structures it holds. It was excavated during the 1994 and 1995 field seasons (Figs. S3-S4).

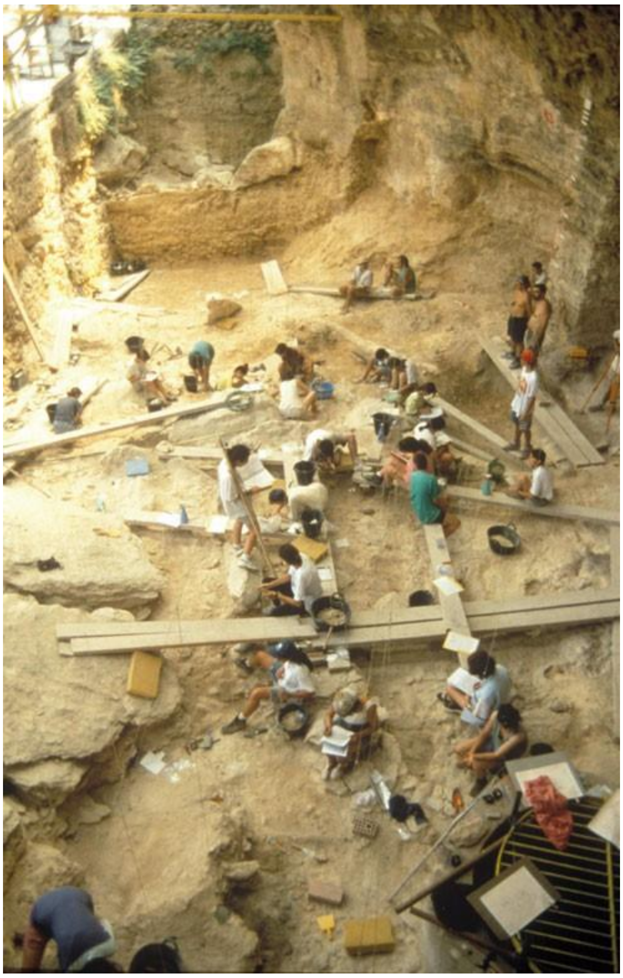

Figure S3. Excavation of level J during the 1994 field season (© IPHES-CERCA. From[6]).

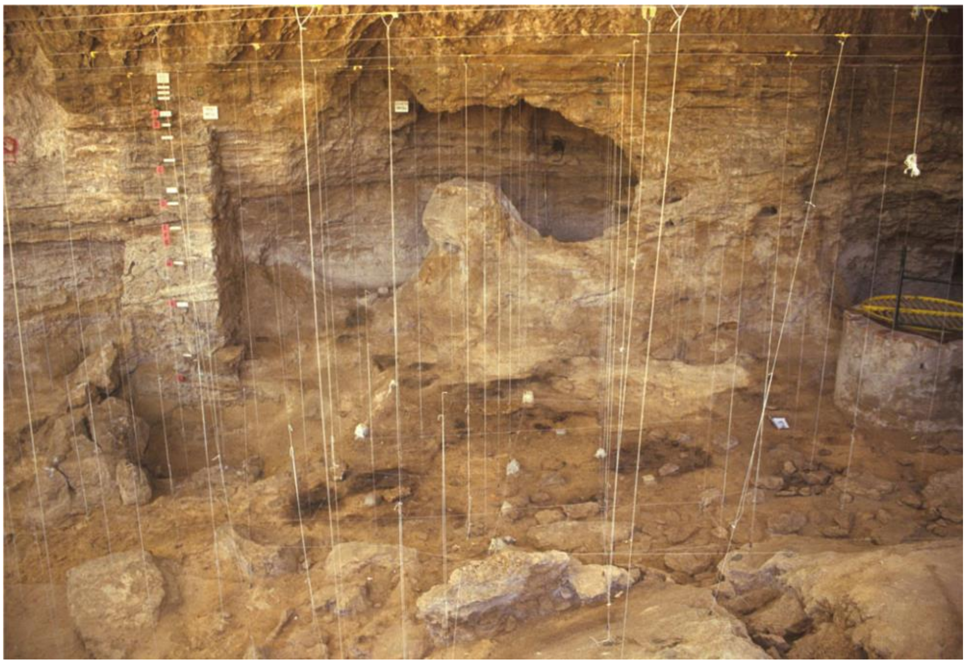

Figure S4. The Abric Romaní during the 1994 field season. The surface shown in the image corresponds to sublevel Ja. © IPHES-CERCA. From[6].

Stratigraphy, sedimentology and paleoambient

Abric Romaní has a 50-metre stratigraphic sequence distributed across 17 distinct archaeological layers[2]. The uppermost stratum has been attributed to the Early Upper Palaeolithic period while the rest of the sequence corresponds to Middle Palaeolithic, from ca. 110 to 40 ka[9–11].

The sedimentary geology of the talus deposits at Abric Romaní exhibits evident zonation caused by the drip line of the rock-shelter roof. In some archaeological levels, like levels J or M, the distance between the base of the shelter wall and the drip line can exceed 12 metres. Therefore, during the periods of maximum growth of the tufa curtain of the rock-shelter roof at Abric Romaní, there was a large surface at the foot of the wall protected from the weather[12]. The sedimentary record at Abric Romaní reveals a sedimentation rate estimated as 0.6 metres/ka. Uranium series dates place the sedimentary succession at Abric Romaní in marine isotopic stage 3 (40 - 60 ka BP)[10,11], although new dating extends this to an age of at least ca. 110 ka BP[9]. Level J is dated by U-series to between 49.3 ± 1.6 ka and 50.4 ± 1.6 ka and by ^{14}C AMS to 47 ka[10].

Through the Coveta Nord stratigraphic profile, five sequences have been established (Fig. S5)[13].

Level J sits in sequence II (Fig. S5). Microfacies analysis of rock-fall and the granular disaggregation of layer J deposits explores the ombrothermic changes in the sedimentary record. The deposits from Level J are described within the framework of the sedimentary process and are made up of wall and tufa roof curtain fragmentation. Microstratigraphic analysis establishes a facies rhythm of 0.75 m–thick lenticular crystalline calcitic gravels (facies 1) and calcitic sand wedges (facies 2). Microfacies within this rhythm show a vertical and horizontal zonality based on the weathering intensity of the rock fragments and calcitic sediments. The sedimentary record evidences decadal to centennial-scale ombrothermic changes based on sedimentary rates close to 1 m/ka in the upper part of sequence II (Fig. S6)[14].

This stratum was divided during fieldwork into two sublevels: Ja and Jb, due to the fact that they were separated by a thin travertine layer.

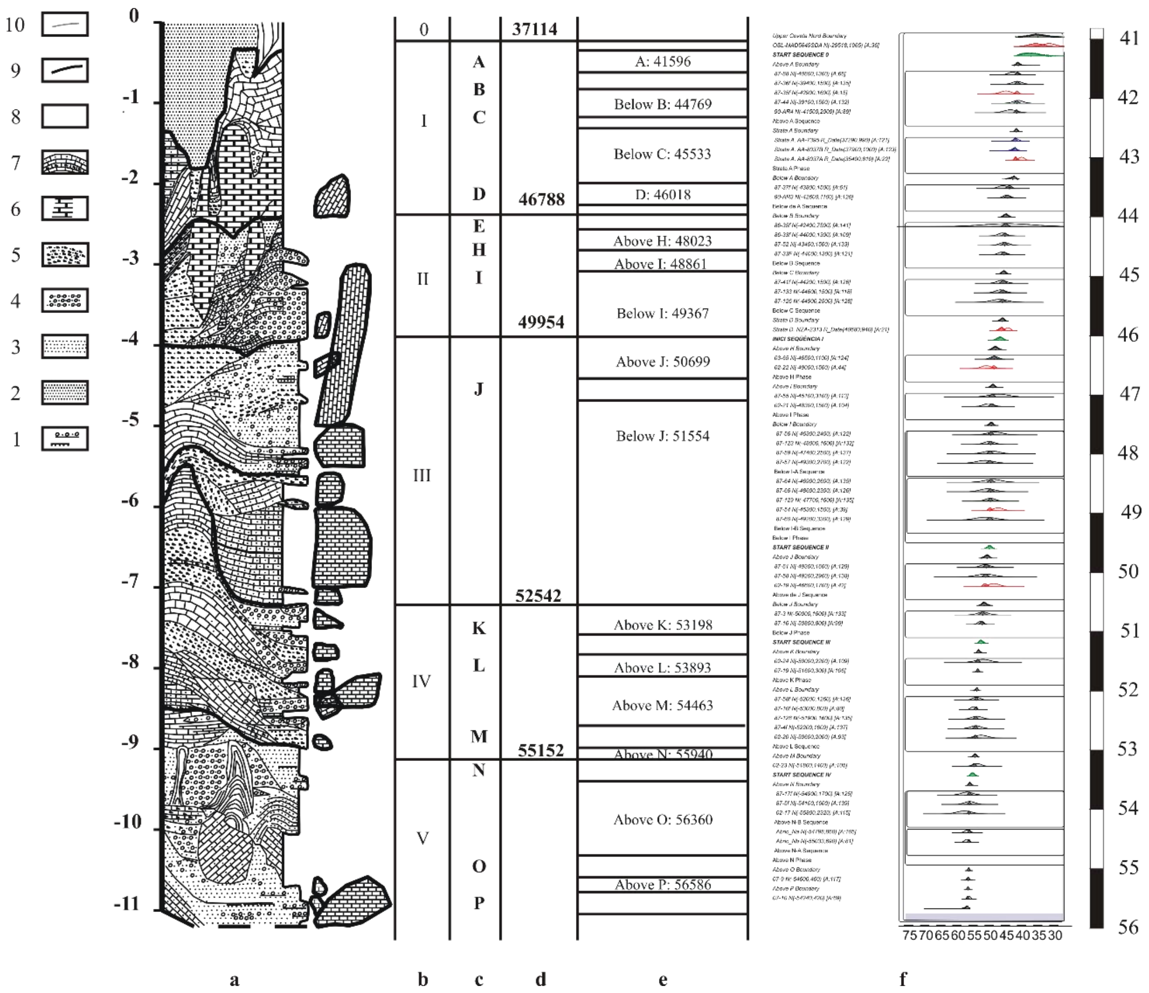

Figure S5. Abric Romaní rockshelter, Coveta Nord Stratigraphy. a, lithostratigraphy, lithofacies legend: 1, bedded calcitic sands and travertinic gravels; 2, siliciclastic and calcitic muddy sands; 3, calcitic sands; 4, travertine gravels and calcitic sands; cristalitic gravels and calcític sands; 6, cristalitic lamination; 7; microbreccia, calcarenite and stromatolithe lamination; 8, archaeological level; 9, boundary sequence; 10, diasteme; b, sequence number; c, letters of archaeological layers: d, bayesian dates for the start of the abric Romani sequences; e, bayesian dates for Abric Romaní. archaeological layers; f, U-series, radiocarbon and luminiscence dates of the Abric Romaní samples.

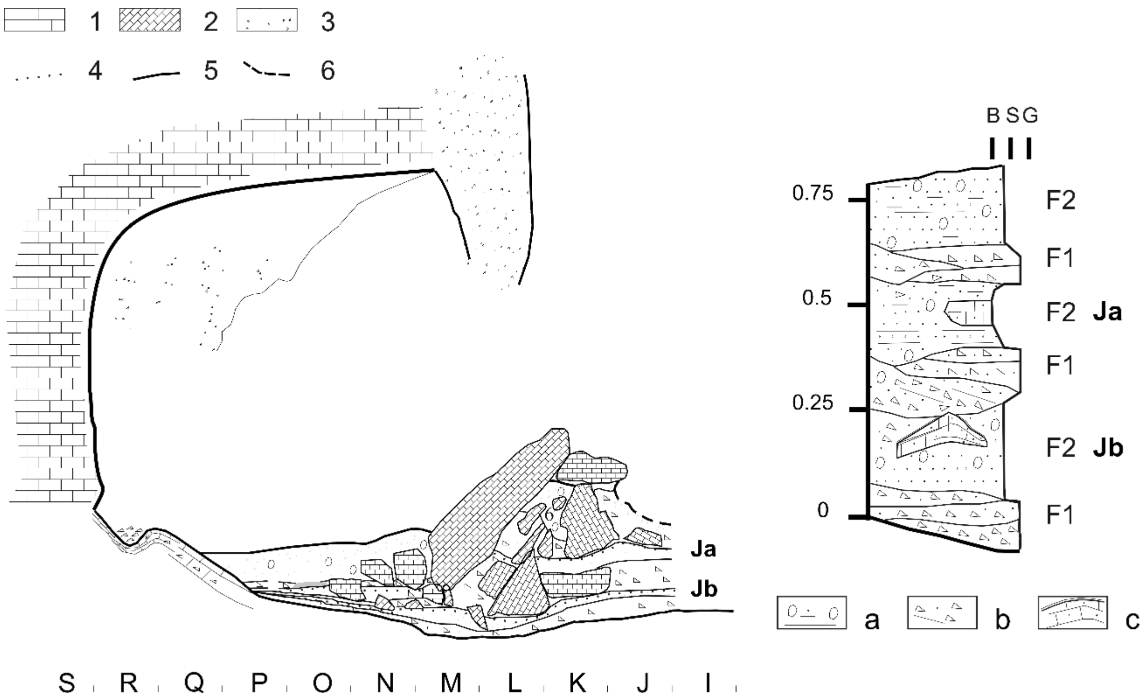

Figure S6. Coveta Nord outcrop and stratigraphic log of archaeological J deposits. Legend: 1, Abric Romaní rock-shelter wall; 2, rocks falls; 3, tufa deposits in the rock-shelter wall and roof curtain; 4, archaeological layers Ja and Jb; 5, stratification surfaces; 6, anthropic truncadure. a, equidimensional fine to medium tufa gravel, calcitic sandy gravel and calcitic muddy sands in horizontal thick bedset; b, laminar cristalline calcitic fine gravel, calcitic sandy gravel stratified in thin bedsets; c, domic to horitzontal tufa deposits made from calcium carbonate cemented phitoclastic or sand in thick laminasets.

The pollen record is divided into 5 biozones showing abrupt climatic changes [15]. Biozones 1, 2, and 3 show variations in tree/non-tree content in a chrono-stratigraphic interval analogous to variations in the oxygen isotopic content determined in Greenland ice core samples. The upper biozones of the sedimentary sequence are more difficult to correlate[15]. The palynological sequence, throughout the different phases, shows predominant *Pinus* characterising the tree formations throughout the period [15,16]. Between 70 and 67 ka BP, the data reflects a warm climate phase with a predominance of arboreal pollens, including *Quercus perenifolios* and *Olea-Phillyrea*. Between 66 and 59 ka BP, grasses predominated, reflecting a colder phase. Between 57 and 50 ka BP, *Pinus* and grasses (*Artemisia* and *Poaceae*) are the most common, with the presence of meso-thermophilic taxa at some moments, indicating climate fluctuations during this period. Between 50 and 47 ka BP, the data reflects a predominance of *Asteraceae*, *Poaceae*, and *Artemisia*, suggesting steppe vegetation with a cold character. Finally, around 46 ka BP, a trend towards a warmer climate is identified with an increase in *Quercus* and *Olea-Phillyrea*[15]. The anthracological data from levels D to O show a predominant taxon in the anthracological assemblage, which is *Pinus sylvestris* type,

representing over 90% of the determinable material[17–19]. The frequency of this species is conditioned by its selection for use as fuel. In level O[19], alongside pine, there are other taxa such as *Prunus* and *Juniperus*, and in level D some fragments of charcoal from mesophilic species such as *Acer*, *Quercus* sp. deciduous and other indeterminate angiosperms could reflect more favourable climate conditions consistent with the palynological data. Red pine forests are the dominant tree formations throughout the sequence, characterised by sparse forests with little taxonomic diversity. The microvertebrates of Abric Romaní reflect a predominance of open-forest species and those that require humidity and colder environmental conditions than at present. In general, all the palaeoenvironmental data from Abric Romaní reflects a mosaic landscape with habitat variability, forests, meadows, riparian forests, and so on, which were more or less extensive depending on the colder or warmer climate phases. Overall, the sequence data reflects colder temperatures than current ones, as demonstrated by the presence of certain micro-vertebrates and the distribution of *Pinus sylvestris* type trees[16].

Archaeological record from level Ja

The archaeological record has provided detailed data, specifically on Neanderthal hunting and animal processing strategies, technology, raw material management, fire technology and spatial organisation.

Lithic assemblage

The lithic assemblage of level J comprises 6,916 artefacts larger than 1 cm (5,446 in sublevel Ja and 1,470 in sublevel Jb). This is one of the largest lithic assemblages of the Abric Romaní sequence, along with levels M, O and Q[20].

Knapping products are the main technological category in the assemblage (flakes: 37% and flake fragments: 50.1%); cores (1.3%) and retouched artefacts (2.8%) are rare (Fig. S7 A to F). In both sublevels, there are generally similar proportions of artefact types. However, there is one clear-cut difference: flakes are more frequent in sublevel Jb, whereas fragments are more common in sublevel Ja.

Expedient discoidal methods predominated during the formation of level J, regardless of the sublevel or the changes in provisioning strategies (Fig. S7F). The core structure is characterised by two opposing flaking surfaces separated by an intersection plane. Some cores present hierarchised patterns with a preferentially exploited flat surface similar to that of typical Levallois cores. The majority show a centripetal scar pattern, like cores derived from recurrent centripetal Levallois modalities (Fig. S7E). In contrast, the

knapping products from level J do not evidence preparation of the flaking surfaces. Lastly, the core-on-flakes identified show both hierarchised and non-hierarchised patterns.

The aim of the core reduction strategies was to produce as many flakes as possible characterised by little concern for blank shape. The distribution of flakes by size classes shows a clear predominance of very small items (61.5% of all flakes; micro-lithic production). The technological attributes of the knapping products are typical of these bifacial centripetal core reduction strategies with a predominance of debordant flakes.

The retouched artefacts are mostly denticulates and notches (Fig. S7 A). Most artefacts (81.2%) show retouching on only one edge and lateral retouching is predominant (73.3%). Retouching is mainly on the longest edge and dorsal retouching is the most widely represented (70.3%). There are no significant differences between sublevels Ja and Jb.[20]

Chert is the most common raw material used, accounting for 80.2% of artefacts in Ja and 90.4% in Jb. Limestone and quartz are also well represented (about 10% each) and were used in reduction sequences (Fig. S7 B, D, H and I). However, limestone primarily appears as unworked nodules and fragments lacking signs of knapping. This is associated with the use of limestone cobbles as hammerstones for other activities[21]. This distinction is notable between sublevels Ja and Jb, with limestone being more common in Ja, not just for non-knapping purposes but also for knapping (Fig. S7 B and H). Other materials like quartzite, porphyry, calcarenite, granite, and sandstone are rare (with less than 3%). They are mostly isolated pieces associated with some single reduction episodes or with non-knapping activities, such as hammerstones for various tasks (Fig. S7C).

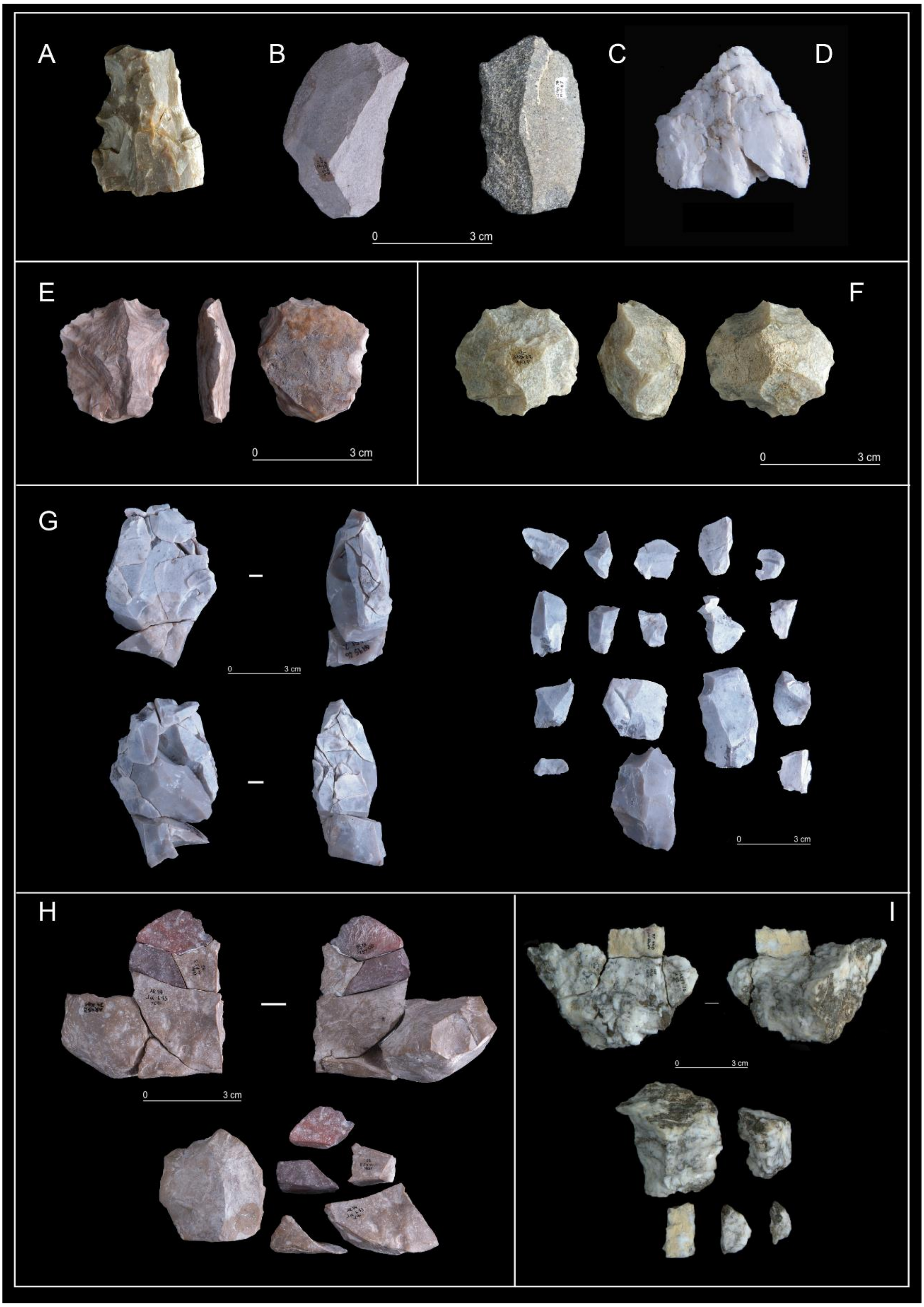

Figure S7. Lithic pieces from Level J: A. Retouched artifact on chert; B. Flake on limestone; C. Flake on quartzite; D. Flake on quartz: E. Hierarchized core on chert; F. discoidal core on chert; G. Example of chert refit comprising 17 pieces; H. Example of limestone refit comprising 6 pieces with varying degree of heat alteration; I: Example of quartz refit comprising 5 pieces. © G. Campeny, M.Vaquero, M.G.Chacón/IPHES-CERCA.

The lithic assemblages of level J indicate two main zones for lithic raw material provisioning. Quartz and limestone can be found within a 5 km radius of the site. Quartz nodules can be located in primary and sub-primary positions in the Palaeozoic slate formations surrounding the site. The cortical surfaces of some types of quartz, the limestone and sandstone indicate a secondary origin in the Anoia river terraces and other colluvial formations. Chert provisioning was mainly from the Ebro basin formations. The second zone, in a range between 5 and 30 km from the site, presents several primary and secondary chert sources. The St. Martí de Tous and Valldeperes chert are the most predominant (~15-20 km). Cortical surface evidence shows exploitation of both primary and secondary sources, with a notable predominance of cobbles from alluvial deposits [20]. These cobbles are very variable in size and shape, influenced by their distance from the primary outcrops. The Level J provisioning suggests an economising strategy in material selection, chert being favoured for its knapping properties, even though it was not the most easily accessible material. The introduction models of the lithic resources are in various forms: 1) entire or almost entire nodules; 2) angular fragments; 3) partially worked cores; 4) single blanks (flakes and retouched artefacts); and 5) sets of blanks from the same reduction sequence (knapped off site). In general, there is a relationship between the introduction and the origin of the raw materials: local materials (limestone and quartz) tend to be introduced as entire nodules, and chert in different ways. These patterns highlight a complex decision-making process related to resource utilisation[21].

The lithic refitting rate from Level J is 10.4. There were 262 refitting groups (chert=185, limestone=54, quartz=20, calcarenite=2, quartzite=1) involving a total of 719 artefacts (Ja=511 and Jb=208) and producing 461 connection lines (62.8% related to the reduction sequences and 36.7% breaks). The mean distance is 251 cm for sublevel Ja and 128 cm for sublevel Jb. The distribution of connection lines according to cardinal direction shows a clear difference between sublevels Ja (NE–SW connections) and Jb (NW–SE directions).

As for artefact categories, the cores had the highest refitting rate (43.4%), followed by fragments (19.1%) and then knapping products (18.8%). Refit analysis confirm the reduction sequences identified through the technological analysis and the raw material provisioning strategies [20,21].

The lithic assemblage at Level J is a palimpsest formed by an unknown number of events. Some of these were associated to knapping episodes, while others were related to the introduction and discarding of single artefacts. Knapping processes in Level J, are characterised by spatial and temporal fragmentation. Chert cores were used primarily, arriving at the site in advanced reduction stages. The assemblage shows no specialised

toolkit; any flake with a sharp edge was a potential tool. Retouched artefacts are rare, with denticulates and notches predominating. The assemblage presents a versatile technology without specialised tools. This is supported by use-wear analysis showing a limited variety of tool use, mainly for butchery activities. The organisation of the lithic production was consistent, utilising expedient methods and producing denticulates and notches for various tasks. This reveals a flexible and versatile approach to lithic tool use and production.

Macrofaunal remains and taphonomy

The faunal remains are very abundant at Abric Romaní. A total of 13 different taxa has been identified, although red deer (*Cervus elaphus*) and horses (*Equus ferus*) are the most common animals at all levels. Aurochs (*Bos primigenius*) and ibexes (*Rupicapra pyrenaica*) are also present in the sequence. The presence of rhinoceroses (*Stephanorhinus hemitoechus*) has also been documented in several levels, although the remains are scarce. From level E, the femur of an undetermined proboscidean was recovered. Despite this diversity, it seems clear that in the Abric Romaní occupations, red deer and horses formed an important part of the Neanderthals' diet. Some carnivore remains have also been documented. These are more abundant in the upper part of the sequence where a more cavernous environment allowed for the association of these animals with occasional dens. Aside from natural intrusions, the remains of *Lynx* sp. and *Felis silvestris*, with cut marks, have been documented in levels B and O, respectively, representing contribution and utilisation[22,23].

The most common taphonomic modifications in the assemblage are related to anthropic activities: cut marks and bone breakage. Bone cremation is very common, although it appears that in many cases it results from indirect and unintentional burning [24]. Carnivore modifications are almost absent in the assemblage, although this type of activity is higher in some levels, such as level K. The anatomical profiles identified at the site are characterised by an abundance of high-survival elements: the skull and mandibles represented particularly by the upper and lower teeth, and fragments of diaphysis of long bones from the extremities; and by the scarcity of low survival elements, the ribs, vertebrae, scapulae, coxae and the extremities of the long bones. Early studies established that this representation was due to the selective transport of prey in function of their weight-sizes. According to this model, red deer were transported whole, and the axial skeleton of larger animals was abandoned at the kill/butchering site. Current research indicates that Neanderthals carried out highly variable transport in function of

the element transported each time, not simply related to the classic *schleep effect* on larger animals, but undoubtedly the nutritional value of the element transported was one of the main factors involved in the selection of the items, especially the marrow content [25,26].

The faunal record at Abric Romaní demonstrates that Neanderthals were active hunters and employed complex strategies for obtaining and transporting the carcasses of large ungulates. The low presence of carnivores and their modifications in the assemblage indicates a low degree of commensalism between the two guilds. The intensive exploitation of carcasses practiced by Neanderthals, combined with their use of fire, probably left few elements that could be scavenged by carnivores.

A total of 6738 faunal remains were recovered from level Ja, with 984 (14.6%) of them being anatomically identified. The level exhibits a predominance of ungulate remains. *Cervus elaphus* (NISP = 497), *Equus ferus* (NISP = 351), *Bos primigenius* (NISP = 88), *Stephanorhinus hemitoechus* (NISP = 33), *Rupicapra pyrenaica* (NISP = 6), *Ursus* sp. (NISP = 1), *Lynx* sp. (NISP = 1), *Canis lupus* (NISP = 2), and *Vulpes vulpes* (NISP = 1) have been identified[27,28]. Carnivore disturbance is extremely rare in the assemblage recorded on 61 specimens. In level Jb, 1,722 faunal remains were recovered: *E. ferus* (NISP = 139), *C. elaphus* (NISP = 96), *B. primigenius* (NISP = 15), *S. hemitoechus* (NISP = 27), *R. pyrenaica* (NISP = 2), *Lynx* sp. (NISP = 1), and *Crocuta crocuta* (NISP = 1) were identified.

Level Ja and Jb represent a long-term occupation, and exhibit similar zooarchaeological features. However, level Ja had a greater spatial distribution; the occupation in Level Jb was concentrated in the central area of the rock-shelter floor.

In the two sublevels, the transport of carcasses of different sizes, and subsequent *in situ* butchery practices are inferred from the presence of cutmarks and anthropogenic bone breakage. These butchery activities encompassed skinning, dismemberment, evisceration, defleshing, and the extraction of tendons and fat. The transport of animal carcasses was variable depending on the elements transported, since elements belonging to all anatomical parts have been recovered, however a trend is observed towards the transport of elements with greater nutritional value, both in terms of the flesh attached and the marrow content[25]. Seasonality studies on ungulates in level Ja have shown that occupations occur at different events throughout the autumn and early winter, usually for a whole season. The mortality profile observed for red deer is situated at the line between attritional and catastrophic profiles, with equal representation of both juvenile and prime adult individuals, albeit with an absence of old animals. In level Jb the

mortality profile of red deer involves exclusively prime adults. This indicates variable prey selection and hunting strategies in the capture of red deer, with mortality profiles that underscore the Neanderthals' procurement of individuals across a spectrum of physical vulnerabilities and strengths over extended temporal intervals, thereby substantiating a pattern consistent with non-selective hunting practices in level Ja, but selective hunting in level Jb. In contrast, the profile of horses involves predominantly prime adults in level Ja and Jb, suggesting the selective hunting of the strongest individuals and, therefore, the animals that could provide the most resources[29]. Burned bone fragments are also present, probably related with hearth management. Post-depositional processes are influenced significantly by low-energy water flows and vegetation. The presence of faunal refits and incipient rounding and polishing demonstrate restricted displacement of the assemblage. Modifications related to the action of plants and water are quite common (Fig. S8).

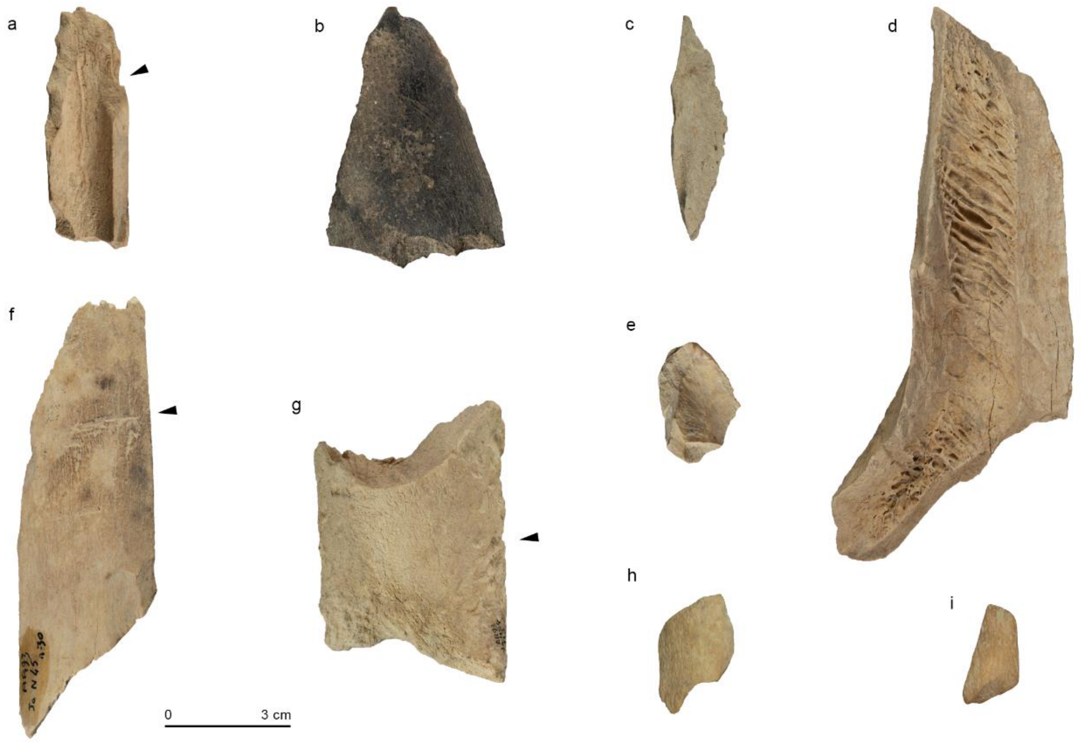

Figure S8. Faunal remains from Level Ja and different modifications. (a) percussion notch, (b) burned bone, (c) medullary bone flake, (d) anthropic bone breakage, (e) cortical bone flake, (f) cutmarks, (g) toothmaks, (h) digested bone, (i) rounding and polishing. © IPHES. M. Guillén/P. Mateo.

Hearths

The record of fire use is further evidence indicating the exceptional preservation conditions of the Abric Romaní archaeological assemblages. Combustion structures have been repeatedly documented on the large archaeological surfaces that have been

excavated, and their spatial documentation allows us to address uncommon issues related to Neanderthal site structure (settlement-size)[2,30–32]. The use of fire is repeated within the shelter, and its spatial documentation allows us to trace analogies of fire use among the different excavated levels and other prehistoric and ethnoarchaeological studies[31]. The study of combustion structures at Abric Romaní raises the question of the existence and number of intra-occupational episodes of each archaeological level and their archaeological visibility for analogy purposes[30–32]. The spatial distribution of combustion structures illustrates the juxtaposition of different fire-use activity areas. Different fire-uses in the combustion structures provide evidence for describing the site structure (settlement size) and demography of archaic human groups[2,31].

To date, more than 400 combustion structures have been documented at Abric Romaní; these involve a diversity of construction techniques. The most frequent combustion structures (> 80%) are flat without stones[12]. There are also flat combustion structures with stones and combustion structures comprising carved concavities and tails containing burnt stones, with small adjacent excavated concavities filled with burnt and unburnt sediments, re-excavated fire pits with burnt and unburnt stones, and so on. In our opinion, many of these combustion structures evidence different uses of fire[12]. The spatial distribution of most flat combustion structures can be described by their distance from the shelter wall according to levels. In Level I, we document combustion structures with accumulations of faunal remains and stone artefacts with no relation to one another [30]. However, there are eight combustion structures, connected by a few lithic and faunal refits that describe a circle. The set of combustion structures in level J shows two modes when quantifying their distance from the shelter wall[14]. Fire use is recorded less than 3 metres from the wall; while, on the other hand, combustion structures are located between 6 and 9 metres away from the wall. The hearths located near the shelter wall or below the dripline have been interpreted as sleeping or multipurpose areas. Level N has been interpreted with the premise that a group of combustion structures[31], one metre away from each other, are the internal part of the settlement in the shelter. This internal area has no stone artefacts or faunal remains, and the combustion structures are located less than 2 metres from the shelter wall. The other combustion structures in level N are distributed in two arcs that are 5 and 9 metres away from the internal area of the settlement. Level O contains a high number of combustion structures[32], some of them are in carved concavities with burnt rocks with very few faunal remains or lithic industry between 6 and 12 metres from the shelter wall. Next to the shelter wall, there are overlapping hearths of large dimensions that contain fire-use focuses separated 1 metre apart, containing faunal remains and lithic tools. Another challenge for archaeological

research is the characterisation of combustion structures that are not associated with lithic artefacts or faunal remains. Among the micro-residues incorporated during fire use determined in the Abric Romaní record are coprolites, calcium oxalates from leaves, fibre residues (leather), pigments, and so on. Thermal modifications, evaluated through the thermal alteration of calcareous constituents and combustion residues rich in charcoal, indicate low-temperature fire use. Carbon-rich sediments contain few ashes. The estimated low temperatures are consistent with observations on the sizes of the fireplaces, or elementary hearths, observed in the microstratigraphy of the combustion structures. Elementary hearths are a mean 0.20 metres in diameter and contain a charcoal rich layer forming a reddened substrate. This size measured in meridional sections of the elementary hearths points to fire use where fuel is limited. There are also combustion structures with large elementary hearths of 0.4 metres diameter, but these are less frequent in the archaeological record of Abric Romaní[31,32]. It is also common to find materials associated with hearths (Fig. S9-10).

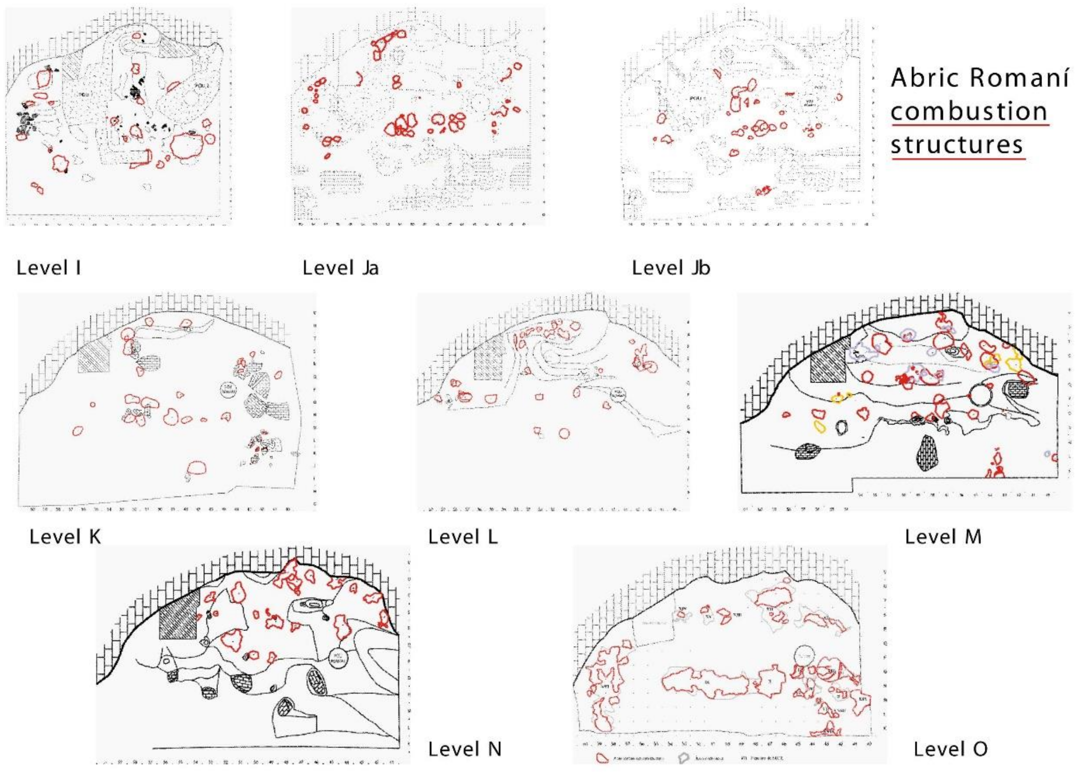

Figure S9. Spatial distribution of combustion structures located at Abric Romaní from level I to level O.

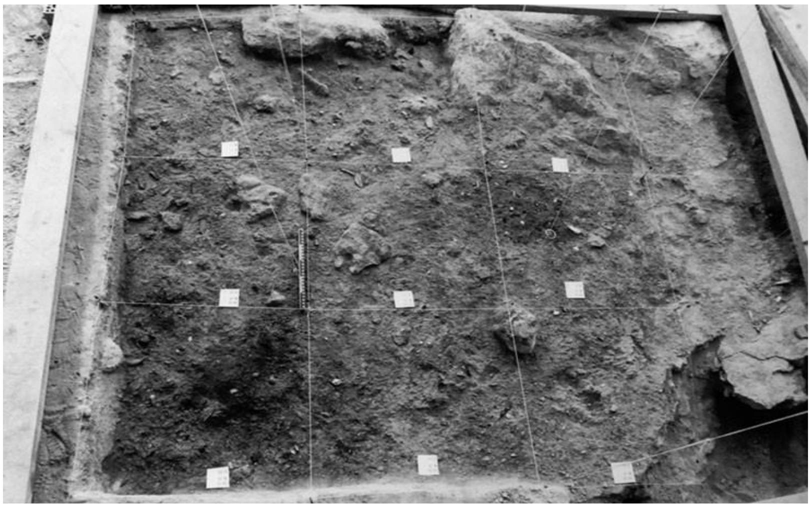

Figure S10. Image of several combustion structures in level Ja (Image from[6]).

A large number of combustion structures (n = 61) have been recorded in level J. Combustion structures have well delimited forms, determined by charcoal-rich sediments, and are related to archaeological remains. The combustion structures in level J can be classified as flat and with or without stones. The combustion structures are stratified in the inner zone because levels Ja and Jb are indistinguishable, forming a micro-palimpsest. In the central zone, these combustion structures present burnt blocks and are microstratified, suggesting an occupational palimpsest[14].

Archaeobotanical assemblage

The archaeobotanical assemblage at Abric Romaní allows the reconstruction of both the palaeoenvironment and anthropogenic activity. It is composed of wood imprints, charcoals and phytoliths. The sequence displays a homogeneous pattern in which *Pinus sylvestris* is the most frequently occurring and abundant taxon, although other levels show a certain variability. Fuel exploitation was probably based principally on dead wood collected locally, along with the intentional accumulation of firewood[19,33].

Level J showcases exceptionally well-preserved plant remnants. *Pinus sylvestris* is the predominant taxon used for firewood. Firewood gathering was dependent on the supplies available in the surrounding environment. Grasses were also used as secondary fuel resources. In addition, the wood imprints indicate that wood was used for purposes beyond simply being firewood, perhaps for manufacturing items[34].

2. Supplementary Information: Extended data about bone spear point found at Abric Romaní

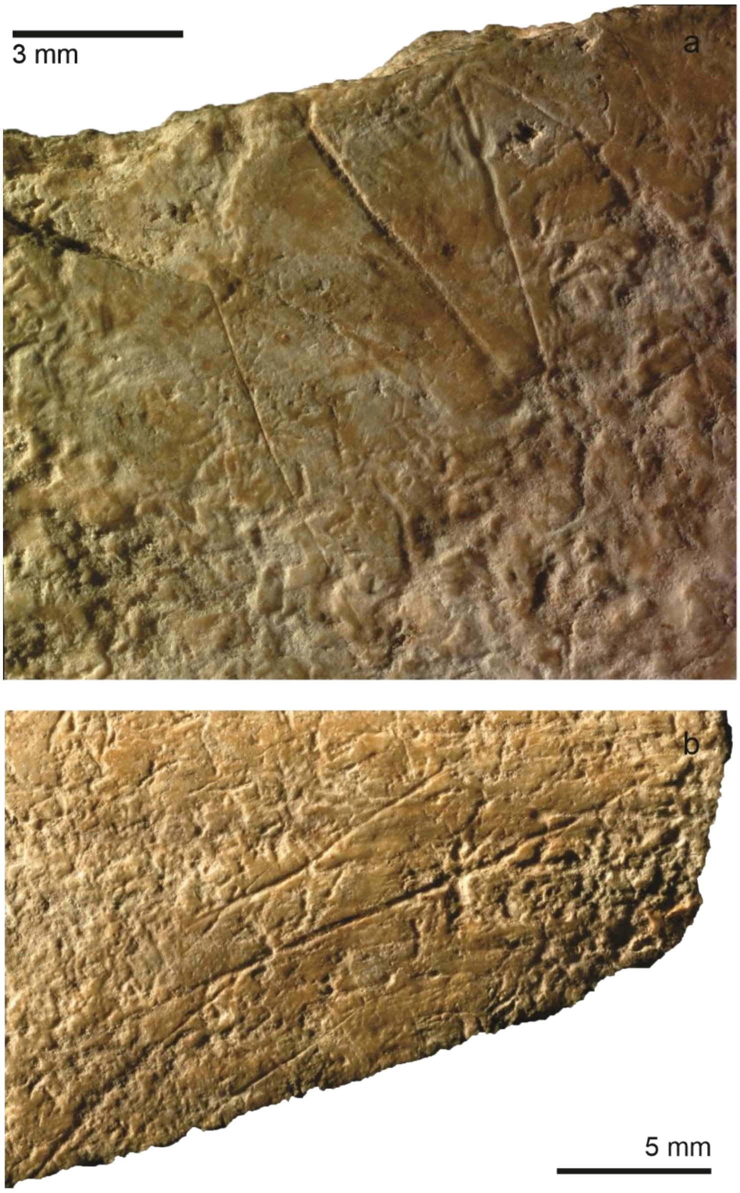

Figure S11. Groups of cut-marks. Scale bars: 3 mm (a), 5 mm (b).

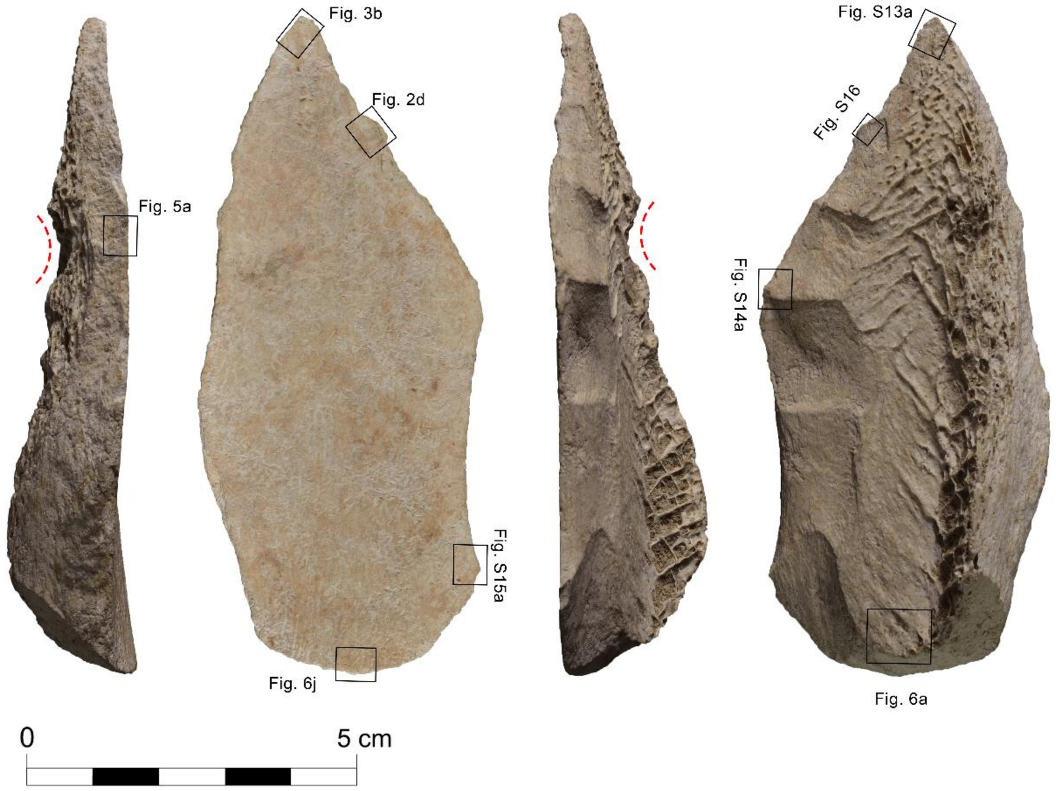

Figure S12. Different views of the bone point from Abric Romaní with localizations of the microphotographs present in the main text. Dotted red lines indicate the position of the groove in the trabecular tissue.

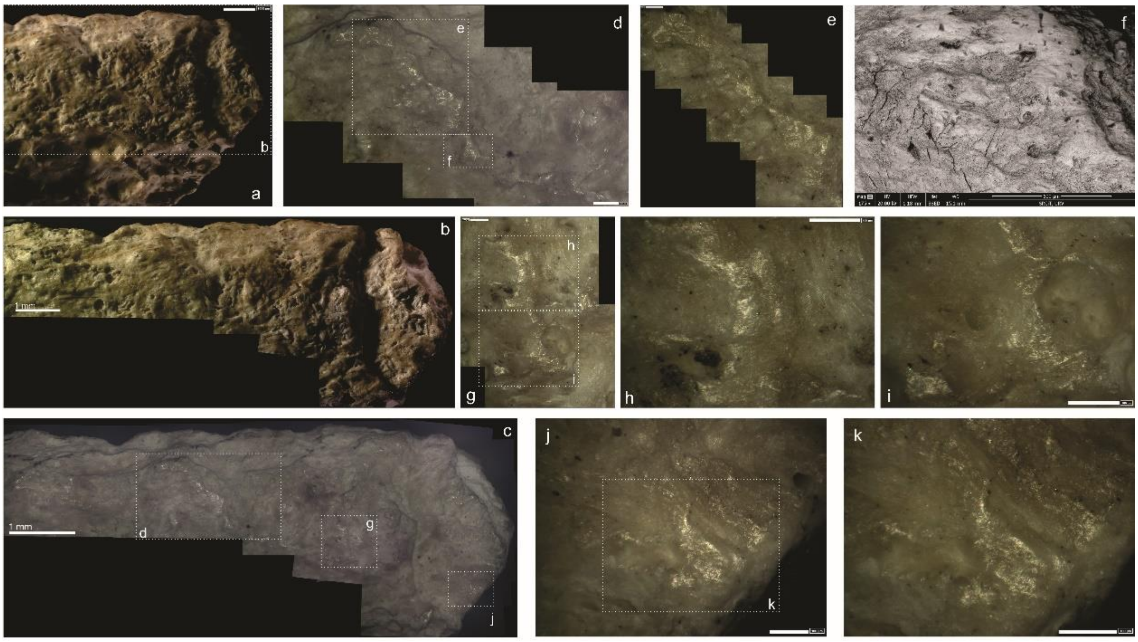

Figure S13. (a) Distal part at medullary surface. (b-c) detail of distal part and localization of use-wear. (c) Detail of distal part, coaxial light. (d-f) Polished area with associated linear marks oriented parallel to the longer axis of the tool. (g-i) band of polished area with associated linear marks. (j) polish located close to the edge. (k) detail of the same area. Images obtained with 3D digital microscope (a-e, g-k), and SEM (f). Original magnification: 35x (a), 140x (b-c), 400x (d, g, j), 600x (e, h, i, k), 175x (f). Scale bars: 1000 µm (a-c), 250 µm (d), 100 µm (e, g-k), 500 µm (f).

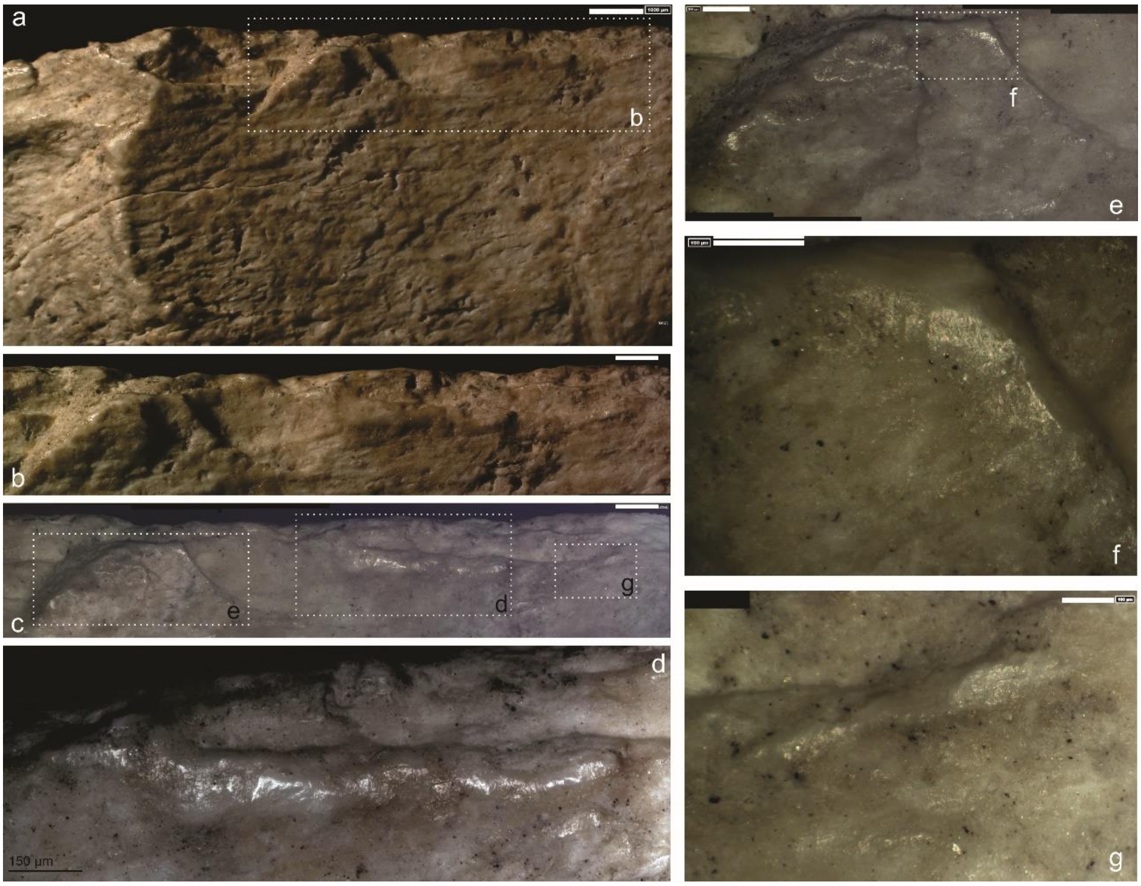

Figure S14. (a) Scarring associated to the left lateral of the tool from the medullary surface. (b,c) Detail of the scarring and localization of other use-wear. (d) Flattened band with polishing and randomly oriented linear marks. (e) Polished areas located at higher points of the microtopography. (f) Slightly rounding at higher points of the microtopography. Linear marks appear perpendicularly oriented to the lateral edge, (g) Incipient flattening. Images obtained with 3D digital microscope (a-c, e-g), and OM (d). Original magnification: 35x (a), 140x (b-c), 100x (d), 200x (e); 400x (f); 600x (g). Scale bars: 1000 µm (a), 500 µm (b, c), 250 µm (e), 150 µm (d), 100 µm (f, g).

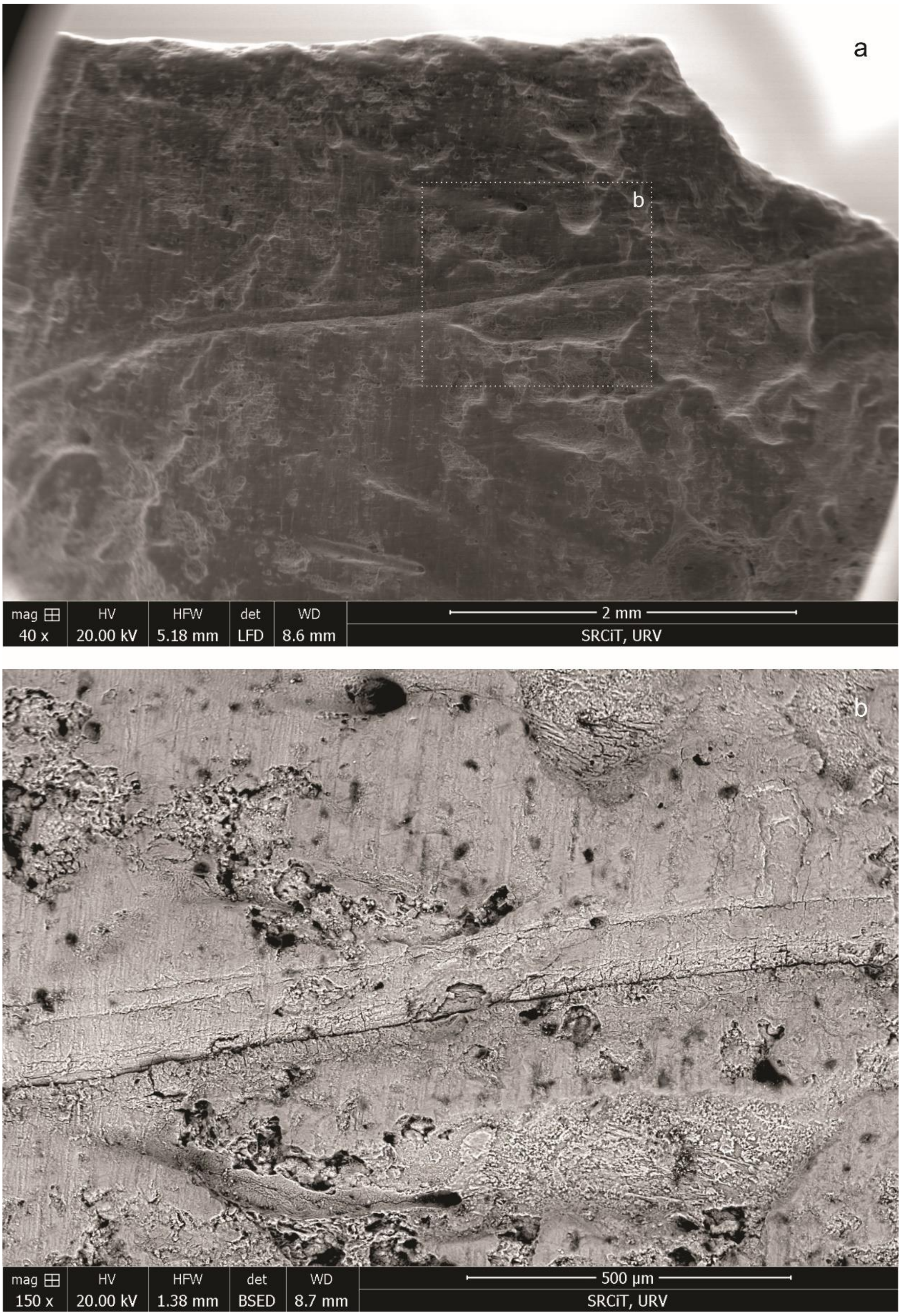

Figure S15. (a,b) Linear marks oriented perpendicular to the lateral edge on the cortical surface. Images obtained with SEM. Original magnification: 40x (a); 150x (b).

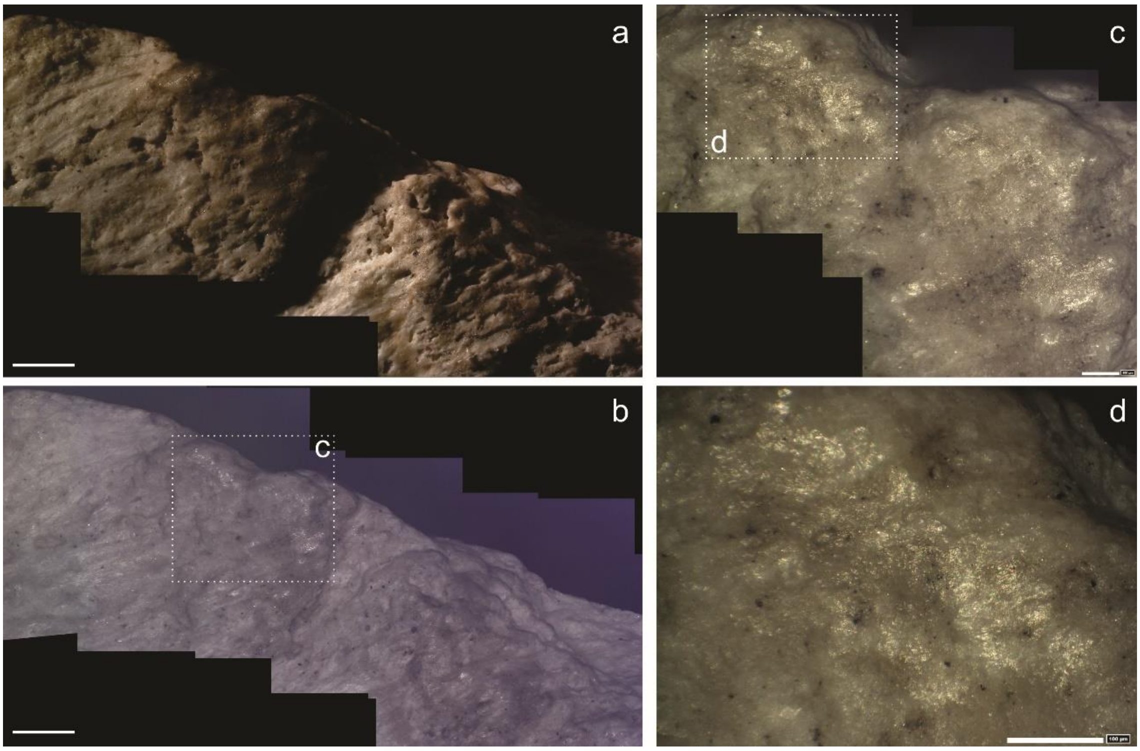

Figure S16. Example of unused surface (a, b). Details of the possible postdepositional microscopic wear. Images obtained with 3D digital microscope. Original magnification: 140x (a, b), 400x (c); 600x (d). Scale bars: 500 µm (a, b), 100 µm (c, d).

3. Supplementary Information: Experimental test

A replicative experiment was performed with 5 bone spear points. The aim was to document the type of impact damages and use-wear left on bones used as thrusting spears, as well as to document possible hafting traces on the tool surface and compare these results with the modifications observed in the purported bone spear point from Abric Romaní.

Bone points

For this purpose, 5 diaphyseal bone fragments were selected from a larger experiment under investigation (partially published in[35,36]). These fragments were obtained by intentional fracture by direct percussion on an anvil of semi-fresh femurs of cattle (*Bos taurus*). The bones were obtained from a local butcher where the carcasses were defleshed. They were then left to dry in the open air for six months (semi-fresh bones). The bones were broken in order to access the marrow and get diaphyseal fragments. The criteria used to select the bone fragments were that they should have a pointed end and a general morphology suitable for attachment and similar to the point from Abric Romaní. Additional shaping of the fragments was avoided to prevent the formation of additional modifications (Fig. S17).

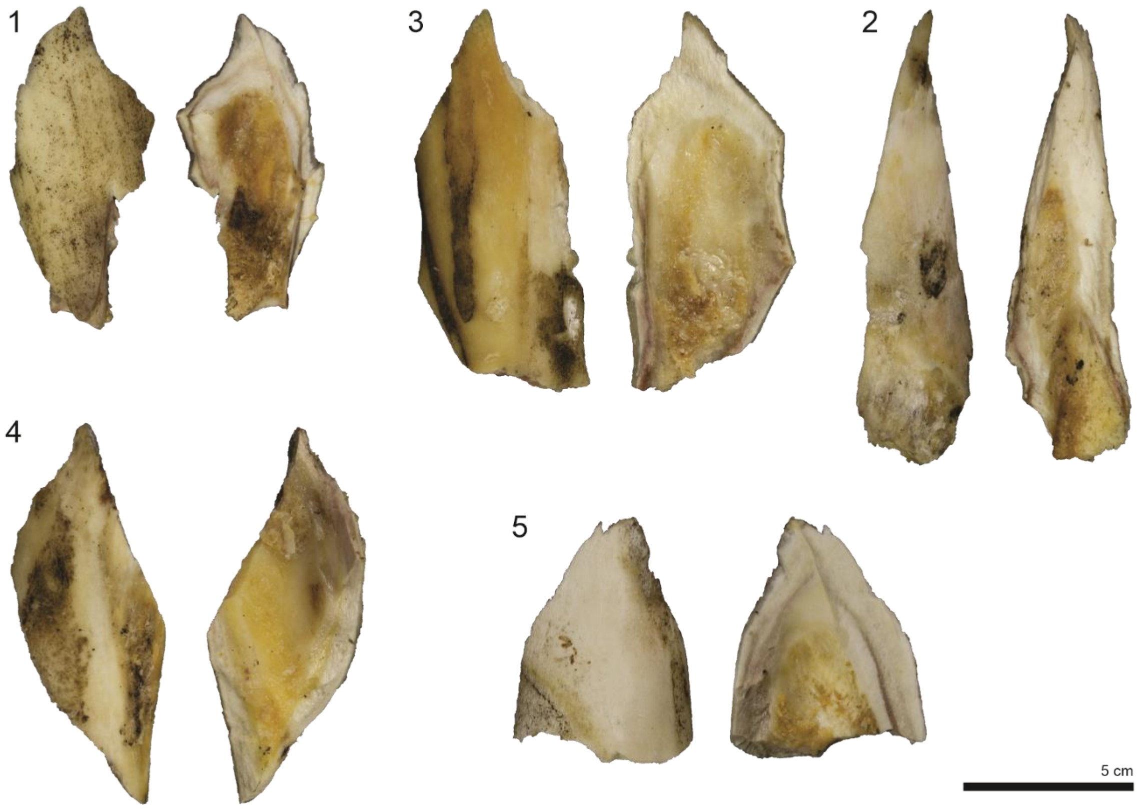

Figure S17. Bone points used in the experiment.

All the fragments were photographed and measured. In addition, several control points were documented with a 3D digital microscope (Hirox KH-8700), distributed across the entire piece, to compare the surfaces of the pointed bones before and after use.

Spears and hafting technique

The hafted spears were replicated using straight green branches of Mediterranean hackberry (*Celtis australis*). The experimental pointed bone fragments were used as tips, hafted in a juxtaposed position onto L-shaped shafts or inserted into a U-shaped housing or male split haft[37,38] (Fig. S18). All points were secured with commercially available hemp rope. Additionally, one was reinforced with adhesive made of pine resin, beeswax and powdered ochre (Table S1). During the assembly process, tip 3 was accidentally dropped and lost a small portion of its tip (compare Fig. S17 and Fig. S18).

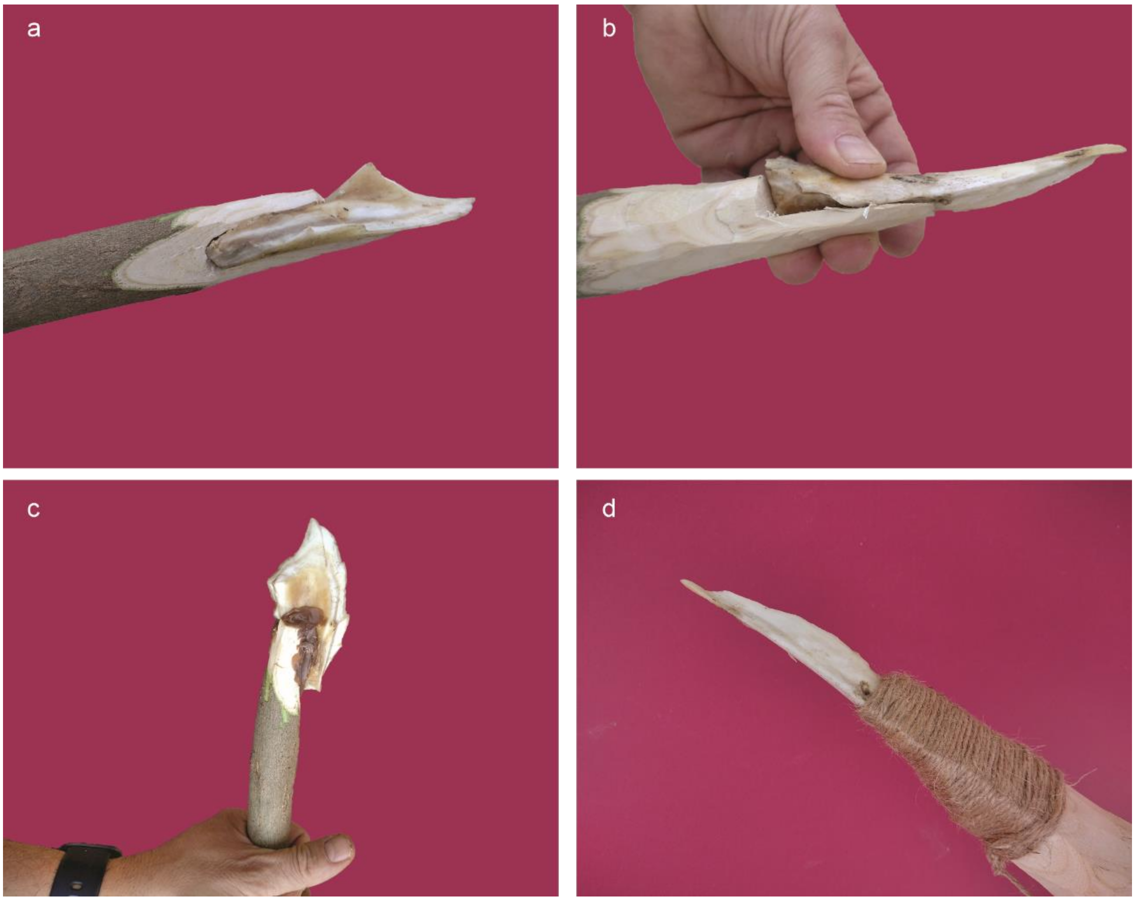

Fig. S18. (a) Example of U-shaped housing or male split haft in point 1. (b) Example of bone point hafted in a juxtaposed position to L-shaped shaft in point 2. (c) and (d) Hafting process.

Spear thrusting

The experimental bone points were utilised at the Boumort National Hunting reserve (Lleida, Spain) where 2 red deer (*Cervus elaphus*) culled by forest rangers to control the

deer population and feed vultures were offered to researchers for experimental or scientific purposes. A deer carcass, placed on a slope, was used as a target. The spears were held with both hands and thrust into the carcass (thoracic and abdominal area). Each attempt was documented with photographs and videos. The condition of the bone tip was examined after each impact. Each point was used until some alteration of the tip was observed (Fig. S19). In cases where it was broken and severely damaged, a new hafted spear was employed (Table S1 and Fig. S20).

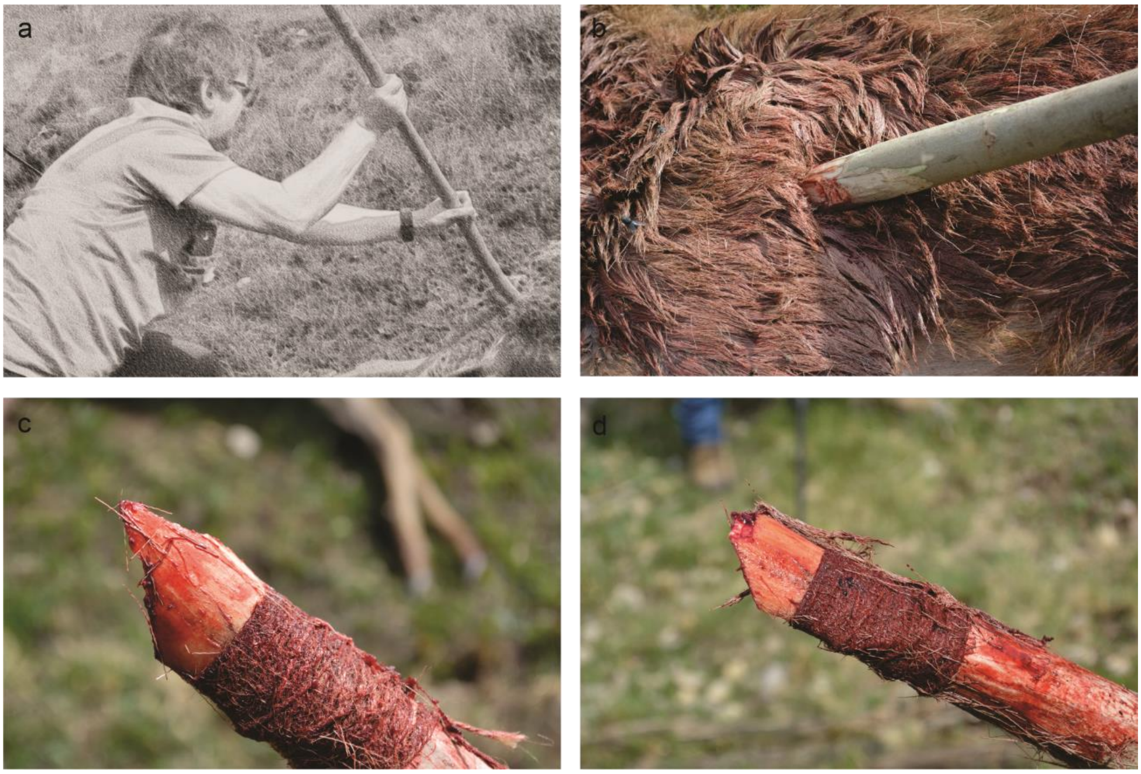

Fig. S19. Experimental framework: (a) Example of spear thrusting. (b) Detail of the impacted area. (c) Bone point (10.2) after the first impact. (d) Bone point after the second and last impact.

	Bone point			Hafted spear				Experimental data				
**ID**	**Length**	**Width**	**Thickness**	**Total length**	**DD**	**CD**	**PD**	**Weight**	**Hafted material**	**Hafting method**	**Impacts (n)**	**Impacted material**
**1**	8.95	4.5	1.56	180	25.4	22.8	20.8	700	adhesive + rope	U-shaped	5	hide
**2**	11.87	3.3	1.46	197	36	29.1	26.7	1360	rope	L-shaped	17	hide+flesh+bone?+soil
**3**	10.52	4.43	1.5	180	34.4	31.2	29	1310	rope	L-shaped	2	hide+flesh+bone
**4**	10.91	4.78	1.95	195	34.9	28	24.1	1150	rope	L-shaped	8	hide+flesh+bone
**5**	6.6	5.1	1.71	194	27.1	21.9	19.2	600	rope	U-shaped	4	hide+flesh

Table S1. Metric characteristics of the bone points and the hafted spear and description of the experimental data. DD: distal diameter. CD: center diameter. PD: proximal diameter. Measures are given in cm and weight in gr. (ID) refers to the nomenclature in a larger ongoing experiment[35,36].

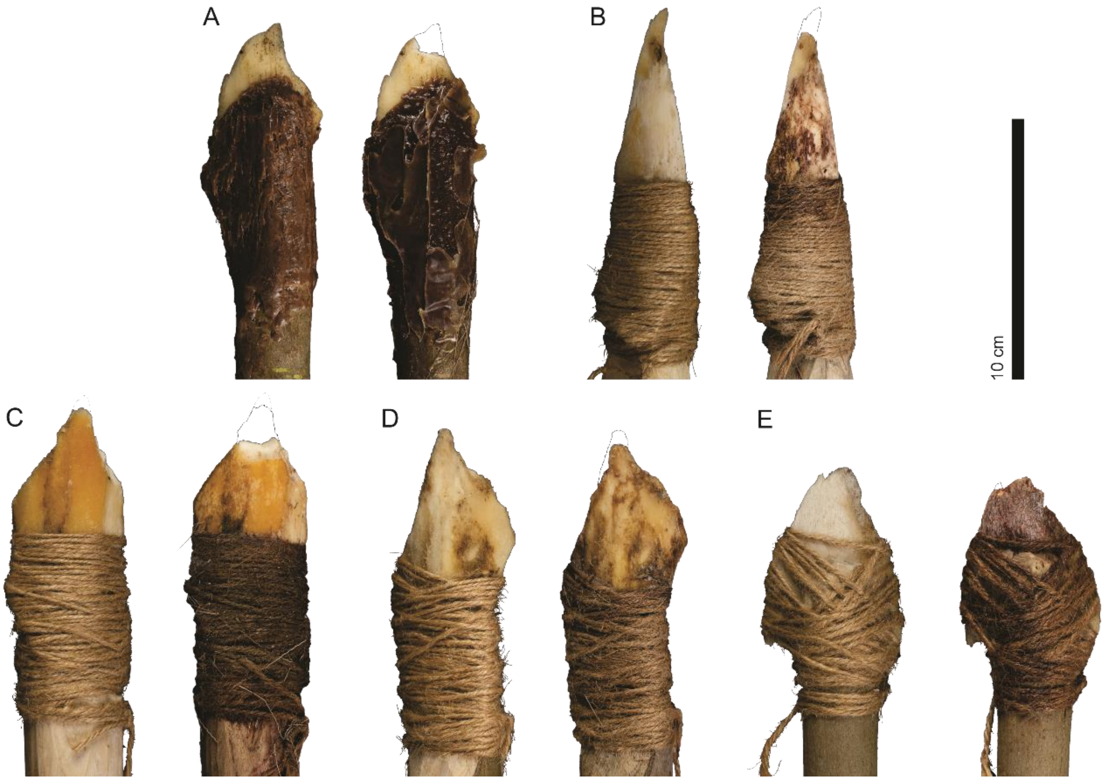

Fig S20. Hafted bone points before (left) and after impacts (right). Solid line: portion lost during use. Dotted line: lost portion during accidental fall.

Functional analysis

After the experiment, the bone points were de-hafted and cleaned. The cleaning protocol consisted of being placed in an ultrasonic bath with a solution of water and lab detergent (Derquim® 2%) as described in Mateo-Lomba et al.[39]. Additionally, the resin was removed with hot water.

Once the pieces had been cleaned, they were photographed and documented using a 3D digital microscope (HIROX KH-8700) and an optical microscope (OM; Zeiss Axio Scope A1) at low and high magnifications (further details in[36]). The tip macro-fractures and microscopic linear impact traces (MLITs) were described according to the terminology in previous works on lithic industry[40–43] and bone industry[44–49]. The hafting traces were identified based on the fact that they showed a heterogeneous topography with flat surfaces at the highest points with random and cross-oriented linear marks of variable width (*vid*. [46]). Wear distribution was documented using the free image stitching software JOIN [50] and the 3D tiling feature of the 3D digital microscope.

Description of the experiment and results

Each of the spears impacted a variable number of times and all of them suffered different types of macrofractures (Fig. S21). Four of the spearheads managed to penetrate the target. Only one of them failed to penetrate the hide. All of these are considered

diagnostic impact fractures[40,45]. No proximal fractures were observed (Table S2, see further details below).

ID	Macroscopic impact fractures	MLITs	Hafting traces
1	Step-terminating transversal bending fracture (Fig. S22A-B)	Presence (Fig. S22C-D)	Presence (Fig. S23)
2	Multiple beveled break (Fig. S24A-B)	Presence (Fig. S24C-I)	Presence (Fig. S25)
3	Hinge-terminating transversal bending fracture (Fig. S27A-B)	Presence (Fig. S27C-F)	Presence (Fig. S28)
4	Step terminating impact burination (Fig. S29A-B)	Presence (Fig. S29C-D)	Presence (Fig. S30)
5	Transversal crushing and incipient bending fracture (Fig. S31)	Presence (Fig. S32)	Presence (Fig. S33)

Table S2. Macroscopic fractures, MLITs and hafting traces observed in the experiment.

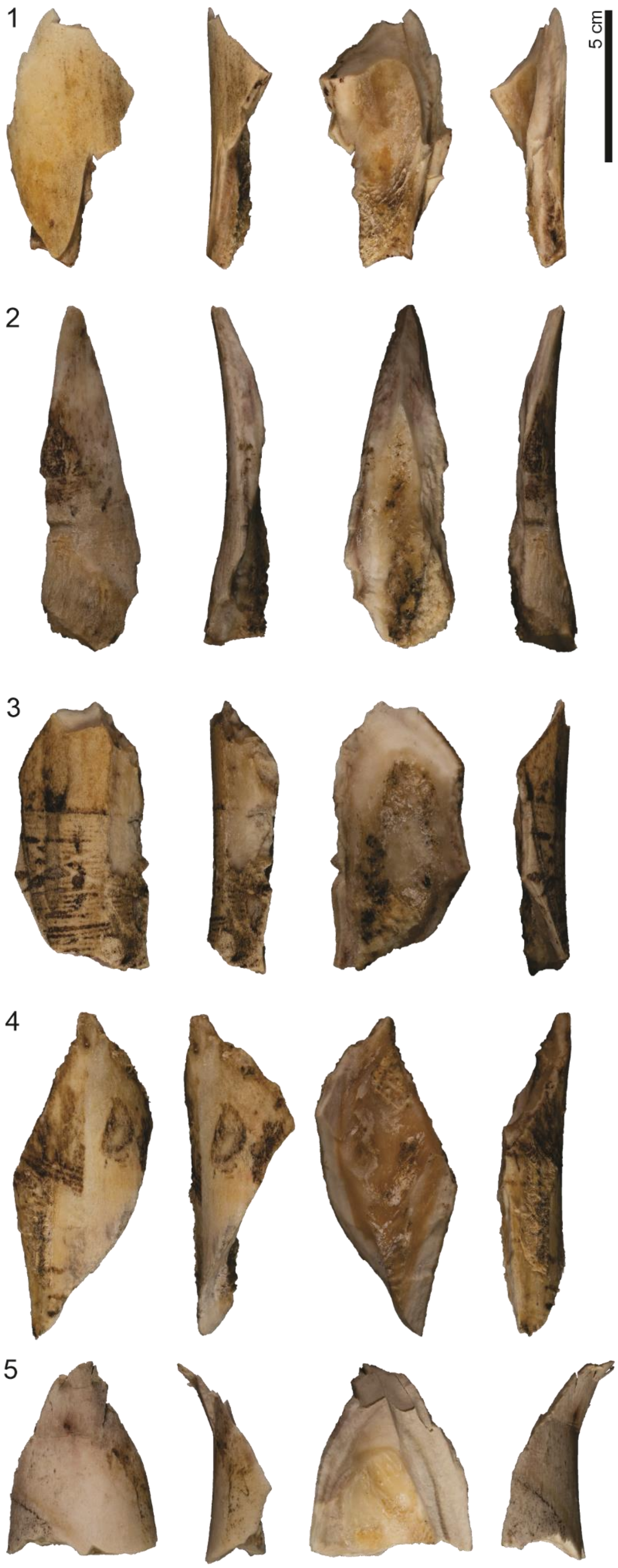

Figure S21. Bone points after using and de-hafting.

Tip 1 hit the target but failed to pass through the hide. The tip shows a transverse bending step fracture. This bending-initiated break was produced in the distal part, initiated from the cortical surface to a fracture plane with a complex termination.

Some use traces could be observed on its surface at the microscopic level. Some polished spots (or MLITs) are present on the cortical surface, on the edge closest to the fracture (Fig. S22).

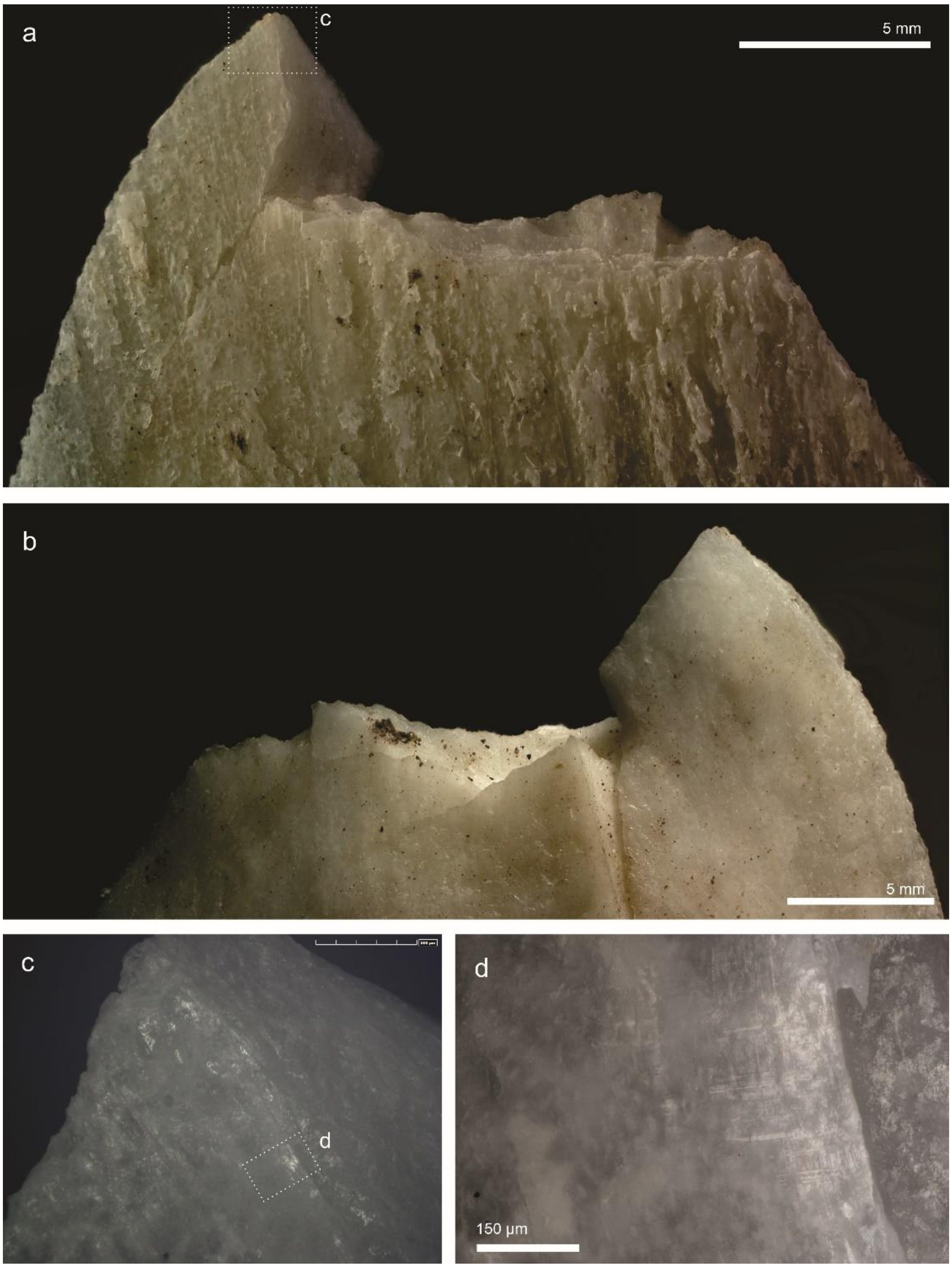

Figure S22. (a and b) Transverse bending step fracture in bone point 1. Some resin residues can be observed on the surface. (c) MLIT visible in association with tip fracture. (d) Detail of the MLIT. Images obtained with 3D digital microscope (a, b), OM (c, d). Original magnification 35x (a, b), 140x (c), 200x (d).

Hafting traces can be identified on the proximal part of the tool. The marks in this case correspond to a polished area with grooves oriented obliquely and parallel to one another. The edge appears slightly rounded (Fig. S23).

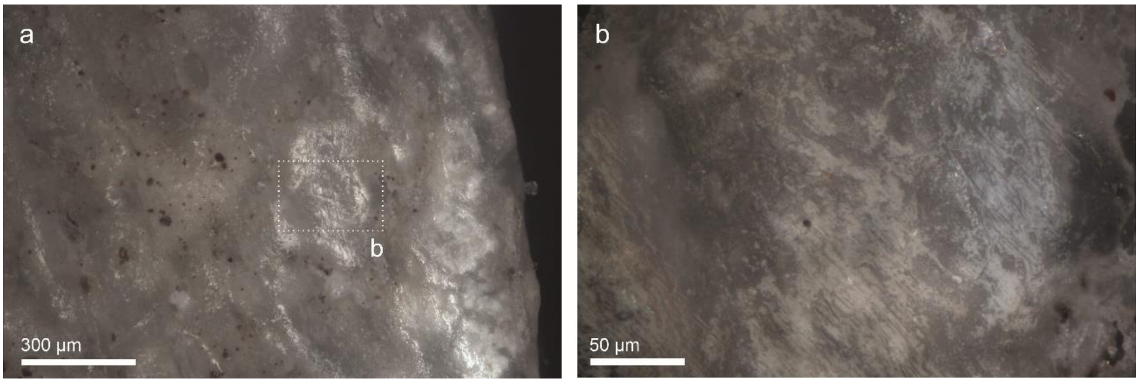

Figure S23. Hafting traces on the lateral side of the bone point (1). (a) Polish and rounding are present in the external part of the active edge. (b) Detail of the same area. Images obtained with OM. Original magnification: 50x (a), 500x (b).

Tip 2 received the highest number of impacts (n=17). It was able to penetrate the target and, on one occasion, missed the target so the tip hit the ground. The fracture observed at the distal end is a multiple bevelled break, initiated on the cortical surface and terminating on the fracture plane surface. Slight rounding is observed on the edge. Moreover, on both cortical and medullary surfaces the polished areas and MLITs appear related to the macrofracture (Fig. S24).

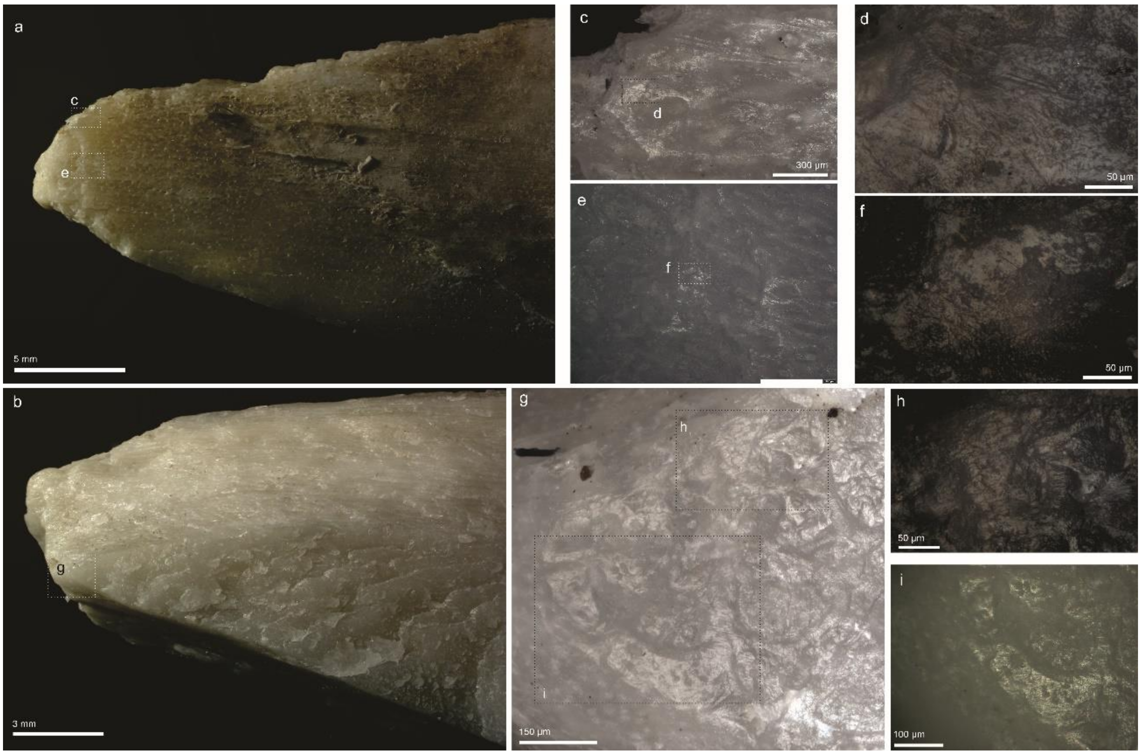

Figure S24. Bone point 2. A: cortical surface of the tip area. B: medullary surface of the tip area. C: MLIT related with the fracture. D: detail of the same area. E: polished surface and MLIT. F: detail of the same area. G: detail of the same area showing rounding of the tip and polishing on the surface. H: detail of the polishing. I: detail of the polishing of the same area. Images obtained with 3D digital microscope (a, b, e, i) and with OM (c, d, f, g, h). Original magnification: 35x (a, b), 50x (c), 500x (d, f, h), 140x (e), 200x (g), 600x (i).

In addition, in the medial part of the point, a flattened area can be observed on the facture plane. At higher magnifications, linear marks can be seen on this surface (Fig. S25).

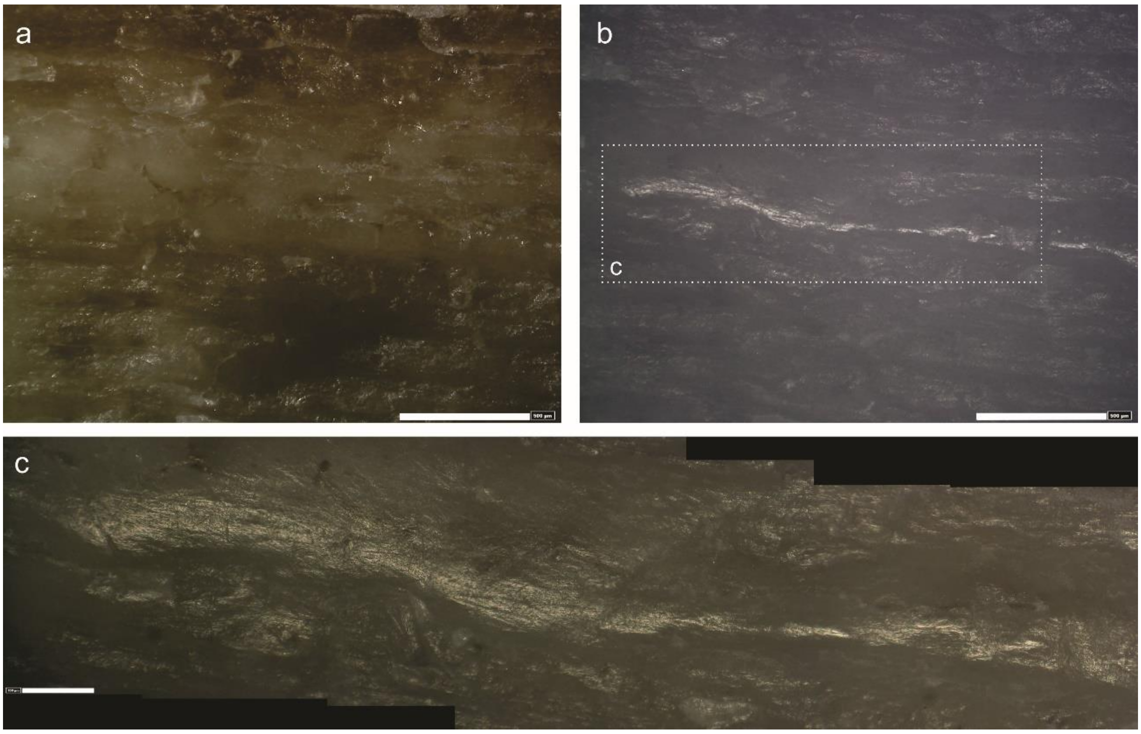

Figure S25. Point 2. A: flattened area on the fracture plane of the point. B: same area viewed with coaxial light. C: detail of the polishing with randomly oriented linear marks. This area shows hafting related use-wear. Images obtained with 3D digital microscope. Original magnification: 140x (a, b), 600x (c).

Tip 3 suffered a small accidental fall that caused a small fragment of its distal end to fracture (see above). During its use as a hafted spearpoint, it was only used in two impacts in which it penetrated the target and struck the left scapula (Fig. S26), suffering a break with a hinge-terminating transverse fracture (Figs. S98). As described above, multiple MLITs appear close to the fracture edge, showing a directionality that coincides with that of the impact (Fig. S27).

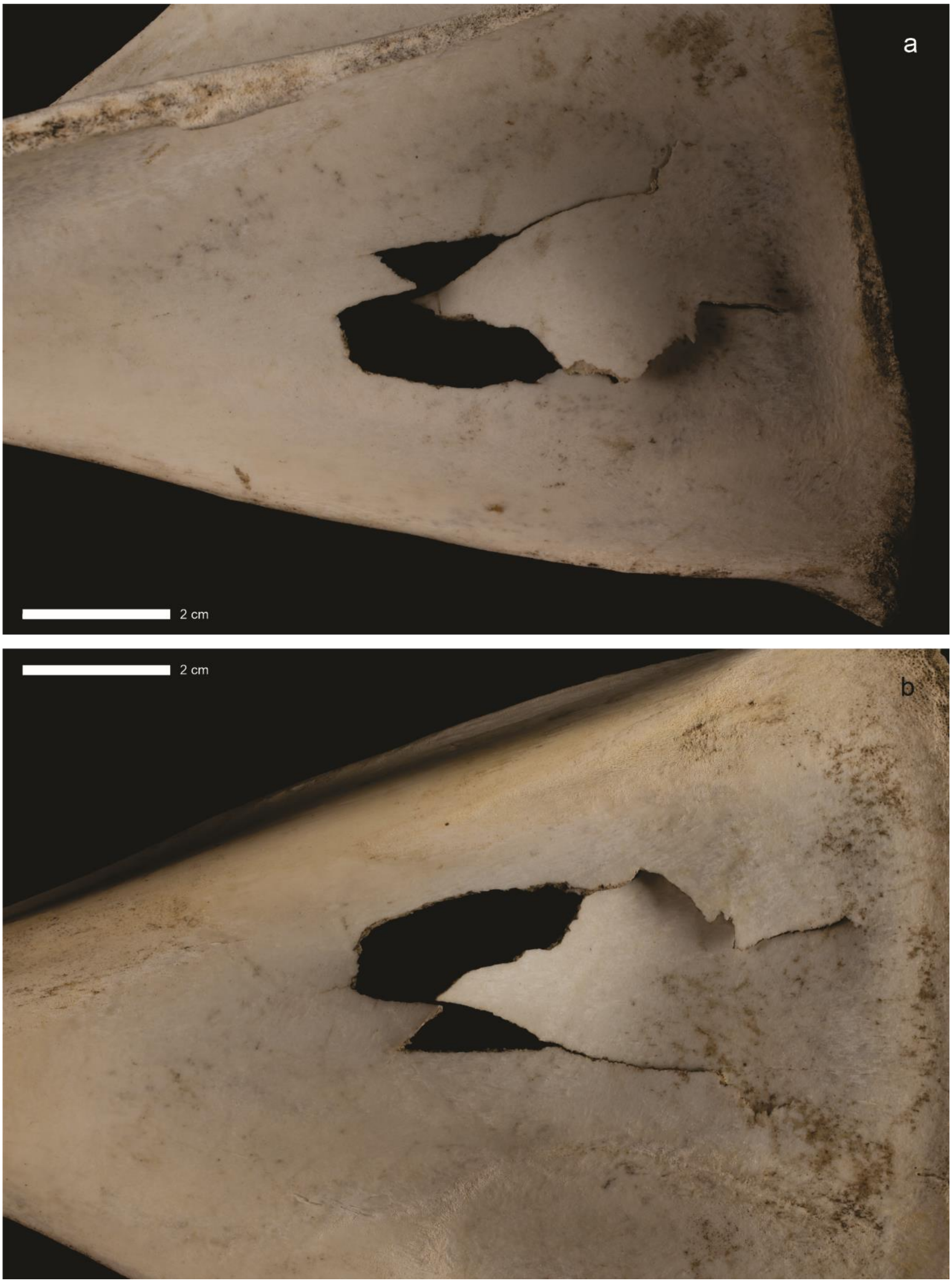

Figure S26. Example of projectile impact damage on a scapula using hafted bone point 3. A: lateral view, B: medial view. Scale bar: 2 cm.

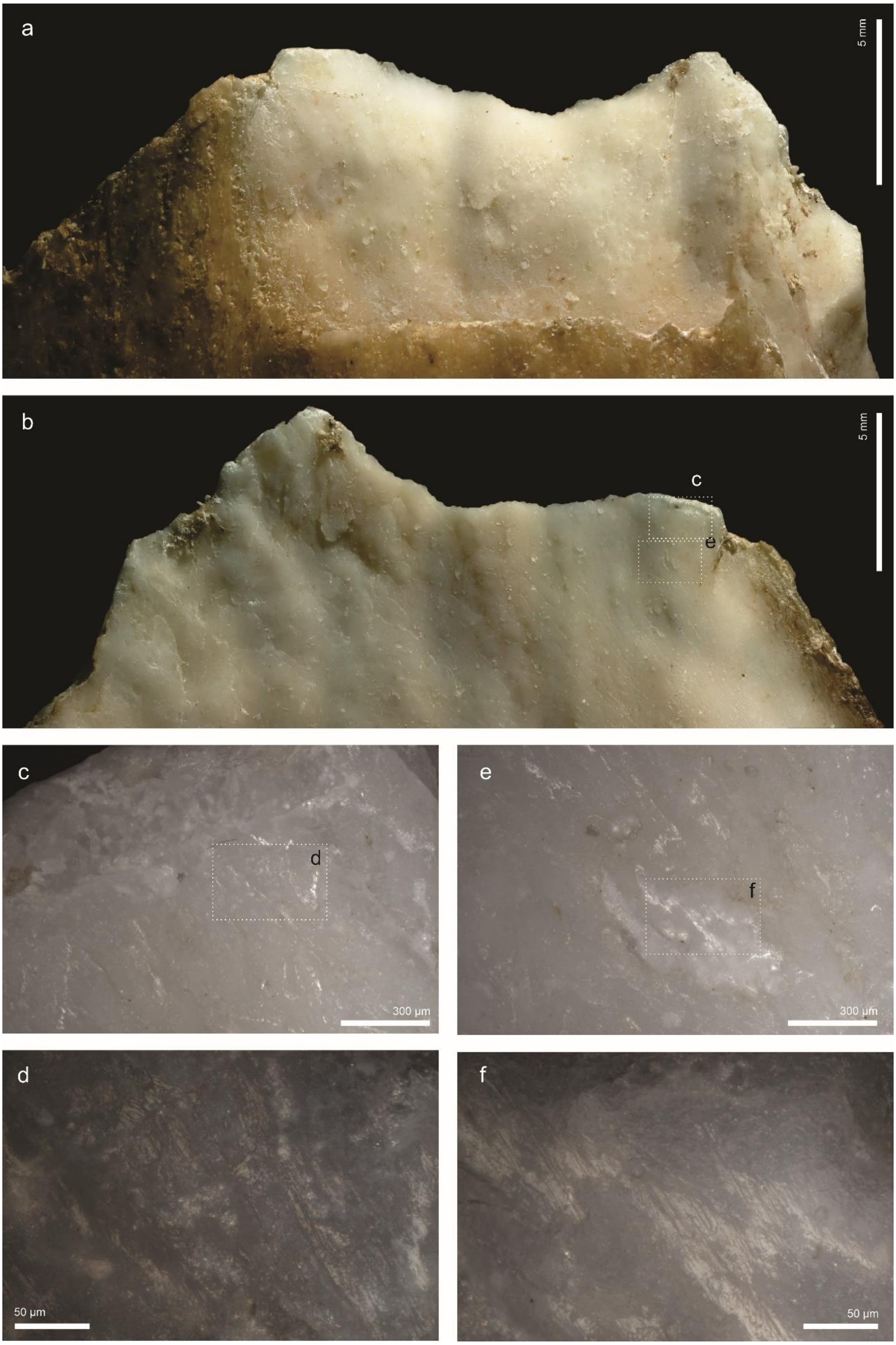

Figure S27. Point 10.2. (a) Cortical surface of the tip. (b) Medullary surface of the tip. (c and e) MLITs related to the contact between the surface of the piece and detaching fragments from macroscopic damage. (d and f) Details from C and E, respectively. The linear marks indicate the

use orientation. Images obtained with 3D digital microscope (a, b), OM (c-f). Original magnification 35x (a, b), 50x (c, e), 500x (d, f).

Hafting traces can be found on the lateral sides of the tool. In this case, the hafting produced polished surfaces on the higher areas of the surface (Fig. S28A-B) and scarring (Fig. S28C-D).

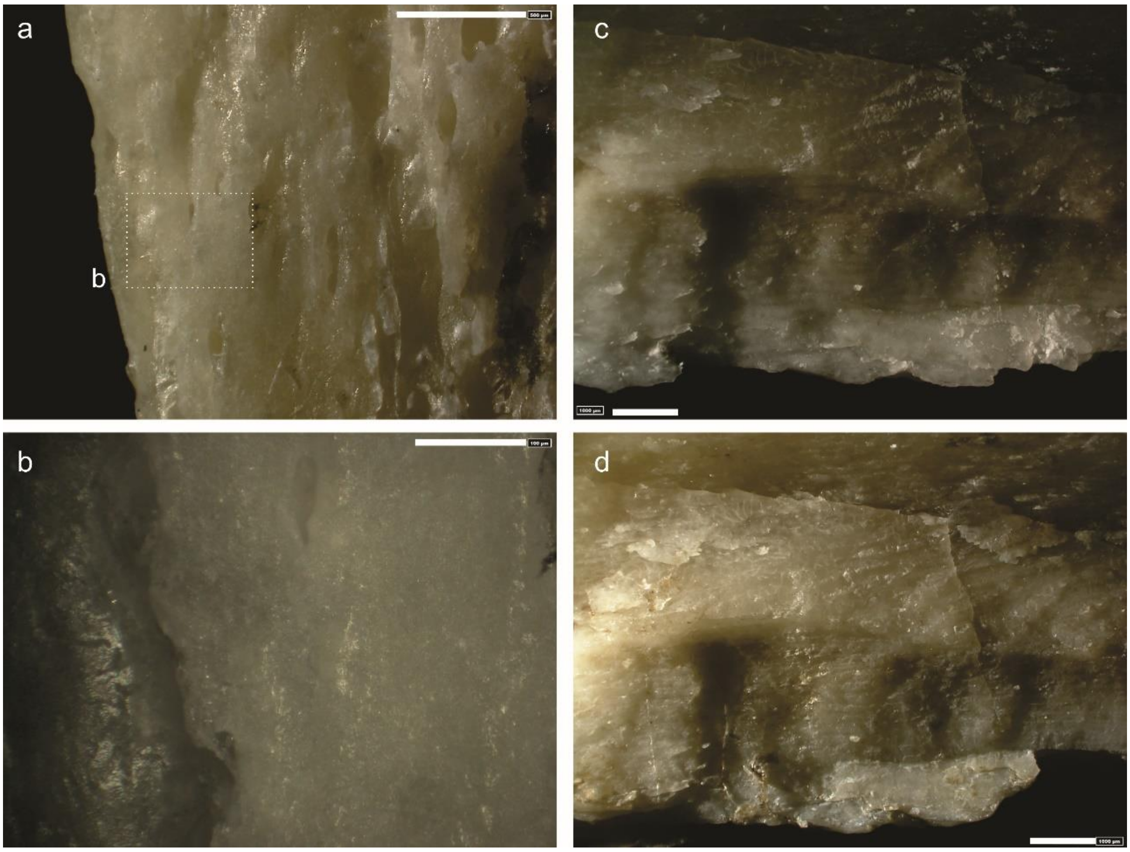

Figure S28. Point 3. (a) Hafting-related use traces. (b) Detail of the same area showing polishing at higher points of the microtopography. (c) Fracture plane before hafting. (d) Scarring is visible on the edge after hafting and using the point as a spear tip. Images obtained with 3D digital microscope. Original magnification: 35x (a, c), 600x (b), 35x (d). Scale bars: 500 µm (a), 100 µm (b), 1000 µm (c, d).

Bone spear 4 suffered a fracture that could be classified as step terminating impact burination, located on the distal part (Fig. S29A-B). The break was initiated on the fracture plane and terminated on the same fracture plane in a feather termination type. MLITs are present on the medullary surface, related with the macrofracturing of the tip (Fig. S29C-D).

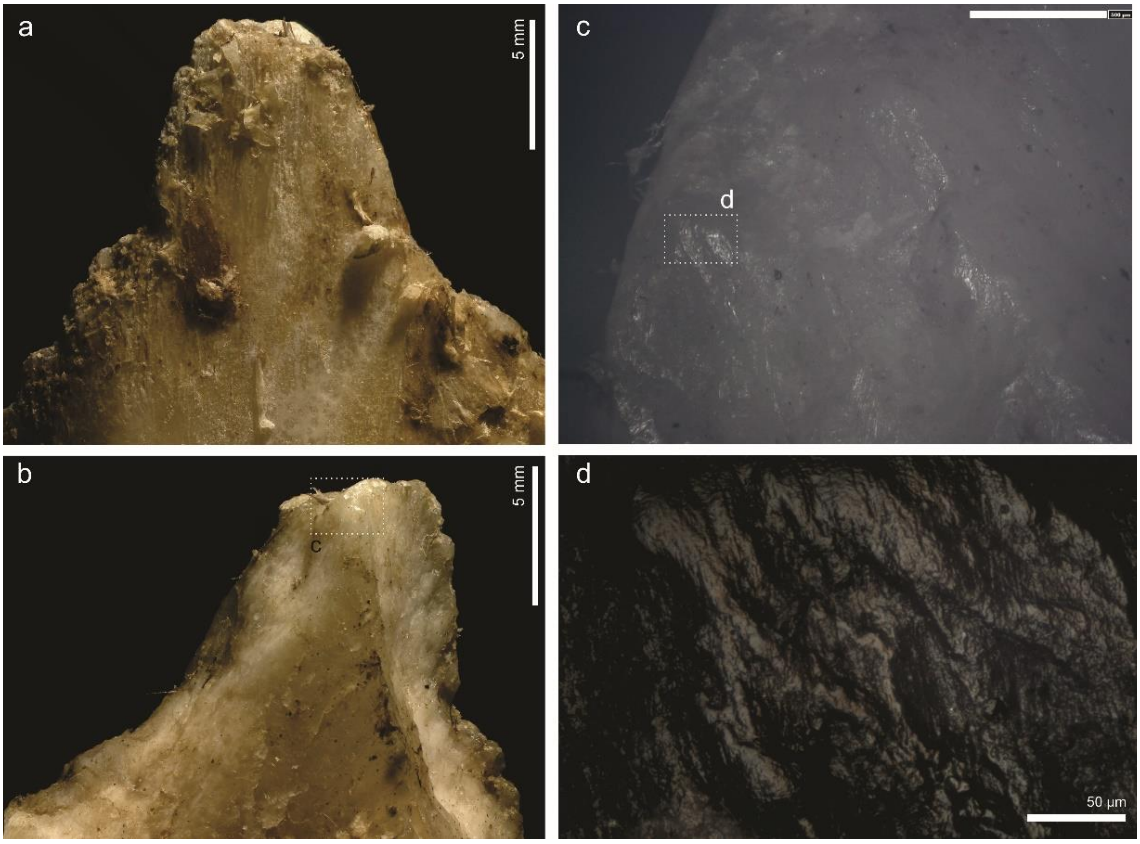

Figure S29. Point 12.3. A and B show the cortical and medullary surfaces of the tip, respectively, after use. (c) Detail of the medullary surface, showing slight rounding and polishing. (d) Detail of the polishing with associated linear marks. Images obtained with 3D digital microscope (a, b and c) and OM (d). Original magnification: 35x (a, b), 140x (c), 500x (d). Scale bar: 500 µm (c).

Hafting traces were identified on the lateral parts of the tool, where the rope covered the surface. The polished points are on the higher parts of the tool. The microtopography does not appear to have undergone any further alteration (Fig. S30).

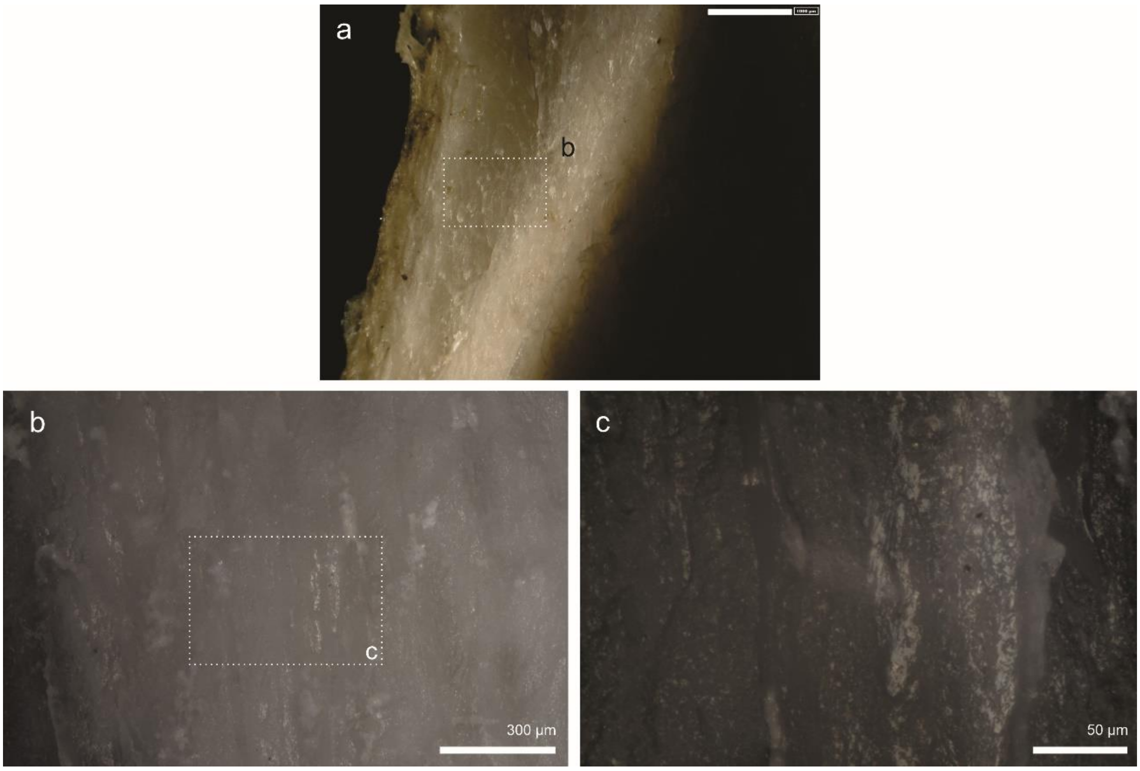

Figure S30. Point 4. (a) Hafting traces on the fracture plane. In this case there is polish on the higher points (b and c). Images obtained with 3D digital microscope (a) and OM (b, c). Original magnification 35x (a), 50x (b), 500x (c). Scale bar: 1000 µm (a).

Tip 5 slightly penetrated the skin of the target on the second impact but on the fourth impact it fractured transversely and longitudinally at the tip, although the distal end did not separate completely from the body of the tool. Only a small portion was lost after use (Fig. S20.5). The macrofracture type corresponds to a transverse crushing and incipient transverse bending fracture (Fig. S31). The termination type is not visible because the tip is still attached to the projectile.

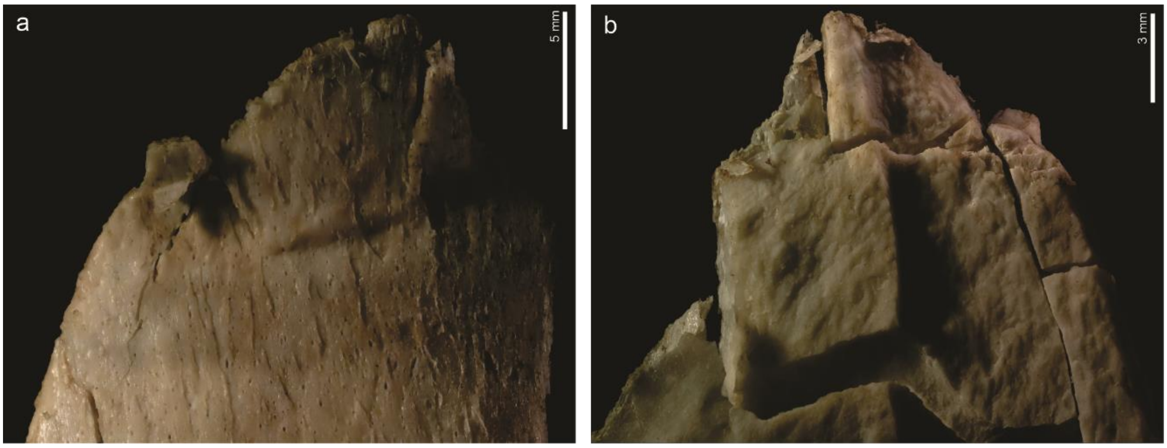

Figure S31. Incipient macrofracture in point 5 (a: cortical surface, b: medullary surface). Images obtained with 3D digital microscope. Original magnification: 35x.

Several MLITs were identified on the medullary surface in an area associated with the fracture (Fig. S32).

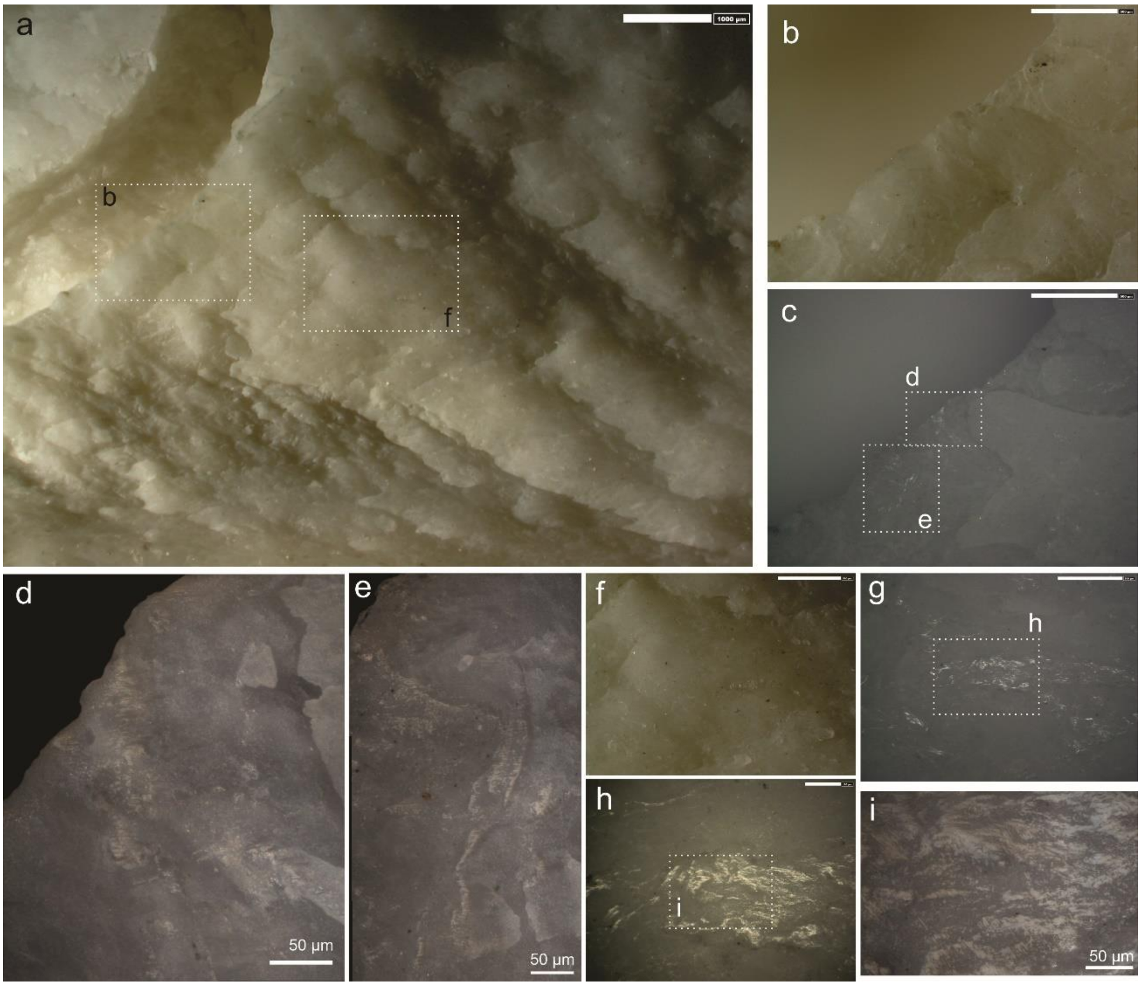

Figure S32. MLITs on the medullary surface of bone point 5. (a) General view of the area and the incipient macrofracture. (b) Location of the use-wear related with the impact. (c) Same point viewed under coaxial light. (d, e) MLITs related to the macrofracturing of the point. Linear marks run perpendicular to the edge indicating the orientation of the action. (f) Detail of the area with use-traces related to use. (g) Same point viewed under coaxial light. (h, i) Details of the MLITs showing polishing and linear marks with the same orientation as explained in D and E. Images obtained with 3D digital microscope (a, b, c, f, g, h) and OM: (d, e, i). Original magnification: 35x (a), 140x (b, c, f, g), 500x (d, e), 600x (h). Scale bars: 1000 µm (a), 500 µm (b, c, f, g), 100 µm (h).

The U-shaped wooden socket of this spearpoint is indirectly recognisable through use-wear. On both sides of the proximal end of the tool there is extensive polishing associated with random and cross-oriented linear marks (Fig. S33). In addition, the corded ligature system is also evidenced on the lateral edges of the points due to the polishing.

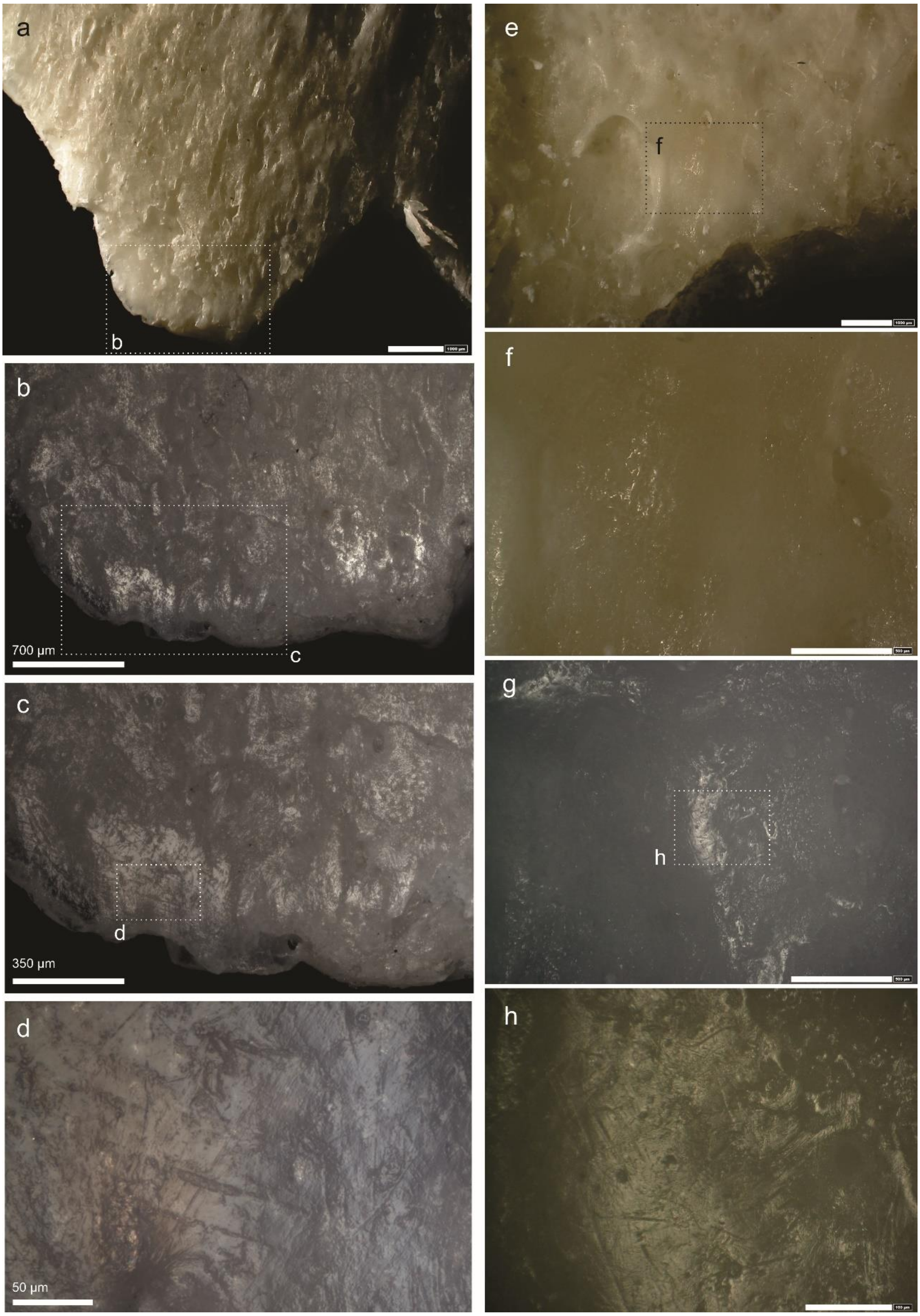

Figure S33. Hafting traces on bone point 5. (a) View from the base of the point on the cortical surface where the shaft went into the socket. (b) Polishing is present in an area close to the edge. (c) Detail of the polishing area with associated linear marks. (d) Details of the linear marks and polishing. The linear marks are randomly oriented and have variable widths. (e) View from the base of the point on the medullary surface, on the opposite side to A, where the shaft went into the socket. (f) Location of the polishing. (g) Polishing viewed with coaxial light, observed at a high point of the microtopography. (h) Detail of the polishing associated with randomly oriented linear

marks. Images obtained with 3D digital microscope (a, e-h) and OM (b, c and d). Original magnification: 35x (a, e), 50x (b), 100x (c), 500x (d), 140x (f, g), 600x (h). Scale bars: 1000 µm (a, e), 500 µm (f, g), 100 µm (h).

Final remarks

Despite the fact that real hunting was not replicated, the experiment shows how useful unmodified bone fragments are for hitting a carcass several times before breaking. Thirty-six impacts were made using the five bone points. This action produced different types of alterations in the projectiles. After use, the most evident damage documented was distal tip fracturing. According to the literature[40,45], some of the different types of fractures observed can be considered diagnostic impact fractures. However, for us it seems that the clearest evidence of the use of a bone point as a projectile is the presence of both macrofractures and microscopic traces associated with the distal fracturing. MLITs were documented in all experimental tips and always appear in association with fractured tips. These results can be added to the scarce experimental reference set on bone projectiles, especially regarding impact and hafting traces at high magnifications [46].

Only one impact-related case of rounding (as in the case of point 2) was observed. This has also been documented in other works[44,46,51–54]. However, tip rounding was not recorded on Bradfield and Lombard's experimental hunting weapons[45].

Although the small experiment described herein was designed to test a hypothesis from archaeological results, it has been possible to replicate some marks produced due to the thrusting impact of the projectile and others related to the hafting. Our objective was never to comprehend the variability of the wear traces formed in impact actions but rather to improve our knowledge of impact and hafting traces and help us interpret a specific archaeological question.

The marks produced have characteristics in common with those identified on the Abric Romaní bone point from level Ja in terms of type, location, distribution and appearance. Further experiments in this field involving more controlled variables are needed in order to evaluate the occurrence and diversity of impact fractures, MLITs and hafting traces on thrusting points.

Ethics statement

The activities performed in the Boumort National Hunting Reserve are developed under the guidelines and supervision of the Reserve and after requesting permission from the Departament d'Acció Climática, Alimentació i Agenda Rural (Generalitat de Catalunya) and its approval. Furthermore, the research is carried out in accordance with the ethical requirements and procedures of IPHES-CERCA, subject to the Code of Conduct of the Institució CERCA of the Generalitat de Catalunya.

SI References

1. Bartolí, R., Cebrià, A., Muro, I., Riu, E. & Vaquero, M. *A Frec de Ciència. L'Atles d'Amador Romani i Guerra*. (Ajuntament de Capellades, Capellades, 1995).
2. Vallverdú-Poch, J., Gómez de Soler, B., Vaquero, M. & Bischoff, J. L. The Abric Romaní site and the Capellades region. in *High Resolution Archaeology and Neanderthal Behavior. Time and Space in Level J of Abric Romaní (Capellades, Spain)* (ed. Carbonell i Roura, E.) 19–46 (Springer, Dordrecht, 2012). doi:10.1007/978-94-007-3922-2_2.
3. Ripoll i Perelló, E. Excavaciones en el abrigo Romaní. *San Jorge* **30**, 14–15 (1958).
4. Laplace, G. Le Paléolithique Supérieur de l'Abri Romaní. *Anthropologie* **66**, 36–43 (1962).
5. Mora, R., Muro, I., Carbonell, E., Cebria, A. & Martínez, J. Chronostratigraphy of 'Abric Romani'. in *L'Homme de Neandertal* 53–59 (E.R.A.U.L, Liège, 1988).
6. Carbonell i Roura, E. *High Resolution Archaeology and Neanderthal Behavior*. (Springer Netherlands, Dordrecht, 2012). doi:10.1007/978-94-007-3922-2.
7. *Abric Romaní, Nivell H: Un Model D'estratègia Ocupacional Al Plistocè Superior Mediterrani*. (Estrat, 5, 1992).
8. Carbonell, E. *Abric Romaní Nivell I. Models d'ocupació de Curta Durada de Fa 46.000 Anys a La Cinglera Del Capelló (Capellades, Anoia, Barcelona)*. (Universitat Rovira i Virgili, Tarragona, 2002).
9. Sharp, W. D. *et al.* Archeological deposits at Abric Romaní extend to 110 ka: U-series dating of a newly cored, 30 meter-thick section. *J Archaeol Sci Rep* **5**, 400–406 (2016).
10. Bischoff, J. L., Julia, R. & Mora, R. Uranium-series dating of the Mousterian occupation at Abric Romani, Spain. *Nature* **332**, 68–70 (1988).
11. Bischoff, J. L. *et al.* Dating of the Basal Aurignacian Sandwich at Abric Romaní (Catalunya, Spain) by Radiocarbon and Uranium-Series. *J Archaeol Sci* **21**, 541–551 (1994).
12. Vallverdú, J. *et al.* Abric Romaní (Capellades, Anoia). in *Pleistocene and Holocene Hunter-gatherers in Iberia and the Gibraltar Strait: the Current*

Archaeological Record (ed. Sala, R.) 221–231 (Universidad de Burgos & Fundación Atapuerca, Burgos, 2014).

13. Vallverdú i Poch, J. Investigación geoarqueológica en los depósitos de ocupación musterienses del Pleistoceno superior del Abric Romaní (Capellades, Barcelona, España). *Boletín geológico y minero* **129**, 129–152 (2018).

14. Vallverdú-Poch, J. & Courty, M. A. Microstratigraphic analysis of level J deposits: A dual paleoenvironmental-paleoethnographic contribution to paleolithic archeology at the Abric Romaní. in *High Resolution Archaeology and Neanderthal Behavior. Time and Space in Level J of Abric Romaní (Capellades, Spain)* (ed. Carbonell i Roura, E.) 77–133 (Springer, Dordrecht, 2012). doi:10.1007/978-94-007-3922-2_4.

15. Burjachs, F. & Julià, R. Abrupt Climatic Changes during the Last Glaciation Based on Pollen Analysis of the Abric Romani, Catalonia, Spain. *Quat Res* **42**, 308–315 (1994).

16. Burjachs, F. *et al.* Palaeoecology of Neanderthals during Dansgaard–Oeschger cycles in northeastern Iberia (Abric Romaní): From regional to global scale. *Quaternary International* **247**, 26–37 (2012).

17. Allué, E. Dinámica de la vegetación y explotación del combustible leñoso durante el Pleistoceno Superior y el Holoceno del Noreste de la Península Ibérica a partir del análisis antrocológico tesis doctoral. (Universitat Rovira i Virgili, 2002).

18. Allué, E. *et al.* Neanderthal Landscapes and Their Home Environment: Flora and Fauna Records from Level J. in *High Resolution Archaeology and Neanderthal Behavior. Time and Space in Level J of Abric Romaní (Capellades, Spain)* (ed. Carbonell i Roura, E.) 135–157 (Springer, Dordrecht, 2012). doi:10.1007/978-94-007-3922-2_5.

19. Allué, E., Solé, A. & Burguet-Coca, A. Fuel exploitation among Neanderthals based on the anthracological record from Abric Romaní (Capellades, NE Spain). *Quaternary International* **431**, 6–15 (2017).

20. Vaquero, M. *et al.* The Lithic Assemblage of Level J. in *High Resolution Archaeology and Neanderthal Behavior. Time and Space in Level J of Abric Romaní (Capellades, Spain)* (ed. Carbonell i Roura, E.) 189–311 (Springer, Dordrecht, 2012). doi:10.1007/978-94-007-3922-2_7.

21. Vaquero, M. *et al.* Time and space in the formation of lithic assemblages: The example of Abric Romaní Level J. *Quaternary International* **247**, 162–181 (2012).

22. Saladié, P. & Aimene, M. Análisis zooarqueológico de los niveles superiores del Abric Romaní (Cataluña): actividad antrópica. in *Actas do 38 Congresso de Arqueologia Peninsular vol. II* 189–201 (ADECAP, Porto, 2000).

23. Gabucio, M. J., Cáceres, I., Rodríguez-Hidalgo, A., Rosell, J. & Saladié, P. A wildcat (Felis silvestris) butchered by Neanderthals in Level O of the Abric Romaní site (Capellades, Barcelona, Spain). *Quaternary International* **326–327**, 307–318 (2014).

24. Téllez, E. *et al.* Incidental burning on bones by Neanderthals: the role of fire in the Qa level of Abric Romaní rock-shelter (Spain). *Archaeol Anthropol Sci* **14**, 1–20 (2022).

25. Marín, J., Saladié, P., Rodríguez-Hidalgo, A. & Carbonell, E. Ungulate carcass transport strategies at the Middle Palaeolithic site of Abric Romaní (Capellades, Spain). *C R Palevol* **16**, 103–121 (2017).

26. Marín, J. *et al.* Neanderthal logistic mobility during MIS3: Zooarchaeological perspective of Abric Romaní level P (Spain). *Quat Sci Rev* **225**, 106033 (2019).

27. Rosell, J. *et al.* A zooarchaeological contribution to establish occupational patterns at Level J of Abric Romaní (Barcelona, Spain). *Quaternary International* **247**, 69–84 (2012).

28. Rosell, J. *et al. Occupational Patterns and Subsistence Strategies in Level J of Abric Romaní*. *Vertebrate Paleobiology and Paleoanthropology* (2012). doi:10.1007/978-94-007-3922-2_8.

29. Marín, J., Saladié, P., Rodríguez-Hidalgo, A. & Carbonell, E. Neanderthal hunting strategies inferred from mortality profiles within the Abric Romaní sequence. *PLoS One* **12**, e0186970 (2017).

30. Vallverdú, J. *et al.* Short human occupations in the Middle Palaeolithic level i of the Abric Romaní rock-shelter (Capellades, Barcelona, Spain). *J Hum Evol* **48**, 157–174 (2005).

31. Vallverdú, J. *et al.* Sleeping Activity Area within the Site Structure of Archaic Human Groups. *Curr Anthropol* **51**, 137–145 (2010).

32. Vallverdú, J. *et al.* Combustion structures of archaeological level O and mousterian activity areas with use of fire at the Abric Romaní rockshelter (NE Iberian Peninsula). *Quaternary International* **247**, 313–324 (2012).

33. Solé, A., Allué, E. & Carbonell, E. Hearth-Related Wood Remains from Abric Romaní Layer M (Capellades, Spain). *J Anthropol Res* **69**, 535–559 (2013).

34. Allué, E., Cabanes, D., Solé, A. & Sala, R. Hearth Functioning and Forest Resource Exploitation Based on the Archeobotanical Assemblage from Level J. in *High Resolution Archaeology and Neanderthal Behavior. Time and Space in Level J of Abric Romaní (Capellades, Spain)* (ed. Carbonell i Roura, E.) 373–385 (Springer, Dordrecht, 2012). doi:10.1007/978-94-007-3922-2_9.

35. Mateo-Lomba, P., Ollé, A. & Cáceres, I. Experimental bone toolmaking: A proposal of technological analytical principles to knapped bones. *Journal of Lithic Studies* **10**, 24 (2023).

36. Mateo-Lomba, P., Fernández-Marchena, J. L., Ollé, A. & Cáceres, I. Knapped bones used as tools: experimental approach on different activities. *Quaternary International* **569–570**, 51–65 (2020).

37. Stordeur, D. Manches et emmanchements préhistoriques: quelques propositions préliminaires. in *La Main et l'Outil. Manches et emmanchements préhistoriques. Table Ronde C.N.R.S. tenue à lyon du 26 au 29 novembre 1984, sous la direction de D. Stordeur* 11–34 (Maison de l'Orient et de la Méditerranée Jean Pouilloux, Lyon, 1987).

38. Knecht, H. Early Upper Paleolithic Approaches to Bone and Antler Projectile Technology. *Archeological Papers of the American Anthropological Association* **4**, 33–47 (1993).

39. Mateo-Lomba, P. *et al.* An assessment of bone tool cleaning procedures in preparation for traceological analysis. *Archaeol Anthropol Sci* **14**, 95 (2022).

40. Fischer, A., Hansen, P. V. & Rasmussen, P. Macro and Micro Wear Traces on Lithic Projectile Points. *Journal of Danish Archaeology* **3**, 19–46 (1984).

41. Odell, G. H. & Cowan, F. Experiments with Spears and Arrows on Animal Targets. *J Field Archaeol* **13**, 195 (1986).

42. Coppe, J. & Rots, V. Focus on the target. The importance of a transparent fracture terminology for understanding projectile points and projecting modes. *J Archaeol Sci Rep* **12**, 109–123 (2017).

43. Metz, L., Lewis, J. E. & Slimak, L. Bow-and-arrow, technology of the first modern humans in Europe 54,000 years ago at Mandrin, France. *Sci Adv* **9**, (2023).

44. Tyzzer, E. E. The "Simple Bone Point" of the Shell-Heaps of the Northeastern Algonkian Area and Its Probable Significance. *Am Antiq* **1**, 261–279 (1936).

45. Bradfield, J. & Lombard, M. A macrofracture study of bone points used in experimental hunting with reference to the South African Middle Stone Age. *South African Archaeological Bulletin* **66**, 67 (2011).

46. Buc, N. Experimental series and use-wear in bone tools. *J Archaeol Sci* **38**, 546–557 (2011).

47. Bradfield, J. & Brand, T. Results of utilitarian and accidental breakage experiments on bone points. *Archaeol Anthropol Sci* **7**, 27–38 (2015).

48. Pétillon, J.-M., Plisson, H. & Cattelain, P. Thirty Years of Experimental Research on the Breakage Patterns of Stone Age Osseous Points. Overview, Methodological Problems and Current Perspectives. in *Projectiles and Hafting Technology* (eds. Iovita, R. & Sano, K.) 47–63 (Springer, Dordrecht, 2016). doi:10.1007/978-94-017-7602-8_4.

49. Pétillon, J.-M. Les pointes à base fourchue magdaléniennes: Approche fonctionnelle. *Préhistoire Anthropologie Méditerranéennes* **9**, 29–55 (2000).

50. Francès-Abellán, A. AnnaFrances8/JOIN-Software-v1: Microscopy image processing program. Preprint at https://doi.org/10.5281/zenodo.8370192 (2023).

51. Arndt, S. & Newcomer, M. Breakage patterns on prehistoric bone points. in *Studies in the Upper Palaeolithic of Britain and Northwest Europe* (ed. Roe, D. A.) 165–173 (Oxford: Archaeo- press (BAR International Series 296), Oxford, 1986).

52. Pétillon, J.-M. *Des Magdaléniens En Armes. Technologie Des Armatures de Projectile En Bois de Cervidé Du Magdalénien Supérieur de La Grotte d'Isturitz (Pyrénées-Atlantiques).* (CEDARC (Artefacts 10), Treignes, 2006).

53. Pokines, J. T. Experimental Replication and Use of Cantabrian Lower Magdalenian Antler Projectile Points. *J Archaeol Sci* **25**, 875–886 (1998).

54. Osipowicz, G., Orłowska, J. & Zagorska, I. Towards understanding the influence of Neolithisation for communities using the Zvejnieki cemetery, Latvia: A technological and functional analysis of the osseous artefacts discovered in the Late Mesolithic burial no 57 and Neolithic burial no 164. *Quaternary International* **665–666**, 65–81 (2023).
